# Whole-Genome Sequencing-Based Re-Identification of *Pseudomonas putida*/*fluorescens* Clinical Isolates Identified by Biochemical Bacterial Identification Systems

**DOI:** 10.1128/spectrum.02491-21

**Published:** 2022-04-07

**Authors:** Mari Tohya, Kanae Teramoto, Shin Watanabe, Tomomi Hishinuma, Masahito Shimojima, Miho Ogawa, Tatsuya Tada, Yoko Tabe, Teruo Kirikae

**Affiliations:** a Department of Microbiology, Juntendo University School of Medicine, Tokyo, Japan; b Department of Microbiome Research, Juntendo University School of Medicine, Tokyo, Japan; c Koichi Tanaka Mass Spectrometry Research Laboratory, Kyoto, Japan; d SUGIYAMA-GEN Co., Ltd., Tokyo, Japan; e BML, Inc., Saitama, Japan; f Department of Clinical Laboratory Medicine, Juntendo University Graduate School of Medicine, Tokyo, Japan; Johns Hopkins Hospital

**Keywords:** *Pseudomonas*, human pathogen, re-identification, whole-genome sequencing, MALDI-TOF MS

## Abstract

The genus Pseudomonas, a complex Gram-negative genus, includes species isolated from various environments, plants, animals, and humans. We compared whole-genome sequencing (WGS) with clinical bacteriological methods and evaluated matrix-assisted laser desorption ionization-time of flight mass spectrometry (MALDI-TOF MS) to identify Pseudomonas species. Clinical isolates (*N* = 42) identified as P. putida or P. fluorescens by a bacterial identification system based on biochemical properties were reexamined by another identification system based on biochemical properties, two systems based on MALDI-TOF MS, and WGS. WGS revealed that 30 of the 42 isolates belonged to one of 14 known Pseudomonas species, respectively. The remaining 12 belonged to one of 9 proposed novel Pseudomonas species, respectively. MALDI-TOF MS analysis showed that the 9 novel species had unique major peaks. These results suggest that WGS is the optimal method to identify Pseudomonas species and that MALDI-TOF MS may complement WGS in identification. Based on their morphologic, physiologic, and biochemical properties, we propose nine novel Pseudomonas species.

**IMPORTANCE** Most of the clinical isolates, identified as P. putida or P. fluorescens, were misidentified in clinical laboratories. Whole-genome sequencing (WGS) revealed that these isolates belonged to different Pseudomonas species, including novel species. WGS is a gold-standard method to identify Pseudomonas species, and MALDI-TOF MS analysis has the potential to complement WGS to reliably identify them.

## INTRODUCTION

Pseudomonas is a complex Gram-negative genus (1) which includes 270 species in the List of Prokaryotic names with Standing in Nomenclature (LPSN) (http://www.bacterio.net). Several Pseudomonas species cause opportunistic infections in humans, including P. aeruginosa (1, 2), P. alcaligenes (3), P. asiatica (4), P. fluorescens (3), P. juntendi (5), P. mendocina (6), P. monteilii (7), P. mosselii (3), P. otitidis (8), P. putida (3), P. stutzeri (3), P. tohonis (9) and P. yangonensis (10). Multidrug-resistant *P. aeuginosa* isolates have been spreading worldwide (2), with resistance also observed in Pseudomonas species, *P. asiatica* (4), *P. juntendi* (5), *P. monteilii* (7), P. putida (11) and *P. yangonensis* (10).

Since P. putida and P. fluorescens groups include 30 and 41 species, respectively, and have 16S rRNA sequences with ≥97% similarity (12, 13); isolates of these groups are often misidentified (14). Recent re-identification of P. putida strains in GenBank using average nucleotide identity (ANI) (15) and digital DNA-DNA hybridization (dDDH) analysis (16) based on their whole genome sequences found that, except for the type strain, many had been incorrectly identified as P. putida (14).

In clinical laboratories, bacteria are routinely identified by automated bacterial identification systems, including the MicroScan WalkAway system (Beckman Coulter, La Brea, CA) and the Vitek 2 system (bioMérieux, Marcy-l'Étoile, France), based on biochemical properties. Recently, matrix-assisted laser desorption ionization-time of flight mass spectrometry (MALDI-TOF MS) low-cost rapid systems, including Vitek MS (bioMérieux) and MALDI Biotyper (Bruker, Billerica, MA), have been adopted for bacterial identification (17). In contrast, whole-genome sequencing (WGS) is not routinely used due to its high cost and complicated procedures. Carbapenem-resistant isolates of *P. asiatica*, a recently proposed Pseudomonas species, had been identified as P. putida spreading in hospitals in Myanmar (4). In this study, we re-identified clinical isolates which had been identified as P. putida or P. fluorescens in clinical laboratories using WGS, and analyzed whole proteins of these isolates using MALDI-TOF MS.

## RESULTS

### Bacterial identification using automated systems routinely used in clinical laboratories.

Of 42 isolates identified as P. putida/*fluorescens* using Microscan Walkaway, Vitek 2 and MALDI Biotyper re-identified 41, and Vitek MS re-identified 38 (Table 1).

**TABLE 1:** Identification results with commercial identification platforms and ANI/dDDH analysis^*a*^

Isolate	Commercial identification platforms				ANI and dDDH analysis
	Microscan WalkAway	Vitek 2	MALDI Biotyper	Vitek MS	
BML-PP010	P. putida/*fluorescens*	Aeromonas salmonicida	*P. marginalis*	P. fluorescens	*P. carnis*
BML-PP011	P. putida/*fluorescens*	P. fluorescens/P. stutzeri	*P. rhodesiae*	P. fluorescens	*P. rhodesiae*
BML-PP012	P. putida/*fluorescens*	P. fluorescens	*P. cedrina*	P. fluorescens	*P. carnis*
BML-PP013	P. putida/*fluorescens*	P. fluorescens	P. viridiflava	Unidentified organism	*P. qingdaonensis*
BML-PP014^T^	P. putida/*fluorescens*	Acinetobacter haemolyticus	*P. koreensis*	P. fluorescens	*P. sputi* sp. nov.
BML-PP015^T^	P. putida/*fluorescens*	P. aeruginosa	*P. nitroreducens*	P. aeruginosa	*P. pseudonitroreducens* sp. nov.
BML-PP016	P. putida/*fluorescens*	P. fluorescens/Aeromonas salmonicida/Acinetobacter haemolyticus	*P. poae*	P. fluorescens	*P. carnis*
BML-PP017	P. putida/*fluorescens*	Acinetobacter haemolyticus	*P. koreensis*	P. fluorescens	*P. atacamensis*
BML-PP018	P. putida/*fluorescens*	P. putida	*P. fulva*	P. putida	*P. fluva*
BML-PP019	P. putida/*fluorescens*	P. fluorescens/P. putida	P. protegens	P. fluorescens	P. protegens
BML-PP020^T^	P. putida/*fluorescens*	P. putida	P. putida	P. putida	*P. parasichuanensis* sp. nov.
BML-PP021	P. putida/*fluorescens*	P. putida	*P. monteilii*	P. putida	*P. juntendi*
BML-PP022	P. putida/*fluorescens*	P. putida	*P. monteilii*	P. putida	*P. asiatica*
BML-PP023^T^	P. putida/*fluorescens*	P. fluorescens/Acinetobacter haemolyticus	*P. koreensis*	P. fluorescens	*P. paraglycinae* sp. nov.
BML-PP024	P. putida/*fluorescens*	P. aeruginosa	*P. koreensis*	P. fluorescens	*P. glycinae*
BML-PP025	P. putida/*fluorescens*	P. fluorescens	*P. corrugeta*	P. fluorescens	P. protegens
BML-PP026	P. putida/*fluorescens*	P. putida	*P. monteilii*	P. putida	*P. juntendi*
BML-PP027	P. putida/*fluorescens*	P. aeruginosa/P. fluorescens/P. putida	Unidentified organism	Unidentified organism	*P. qingdaonensis*
BML-PP028^T^	P. putida/*fluorescens*	Burkholderia gladioli	P. plecoglossicida	P. putida	*P. ceruminis* sp. nov.
BML-PP029	P. putida/*fluorescens*	P. stutzeri	P. otitidis	Unidentified organism	P. otitidis
BML-PP030^T^	P. putida/*fluorescens*	P. aeruginosa	*P. koreensis*	P. fluorescens	*P. parakoreensis* sp. nov.
BML-PP031	P. putida/*fluorescens*	P. fluorescens	*P. rhodesiae*	P. fluorescens	*P. rhodesiae*
BML-PP033	P. putida/*fluorescens*	P. aeruginosa/P. putida	P. otitidis	Unidentified organism	P. otitidis
BML-PP034	P. putida/*fluorescens*	P. mendocina	*P. nitroreducens*	P. aeruginosa	*P. pseudonitroreducens* sp. nov.
BML-PP035	P. putida/*fluorescens*	Unidentified organism	*P. rhodesiae*	P. fluorescens	*P. carnis*
BML-PP036^T^	P. putida/*fluorescens*	P. fluorescens/Acinetobacter haemolyticus	*P. atoformaus*	P. fluorescens	*P. pharyngis* sp. nov.
BML-PP037	P. putida/*fluorescens*	P. stutzeri	Pseudomonas *sp.*	P. stutzeri	*P. tohonis*
BML-PP038	P. putida/*fluorescens*	P. fluorescens/Aeromonas salmonicida/Acinetobacter haemolyticus	P. fluorescens	P. fluorescens	*P. carnis*
BML-PP039	P. putida/*fluorescens*	P. fluorescens	*P. koreensis*	P. fluorescens	*P. glycinae*
BML-PP040	P. putida/*fluorescens*	P. fluorescens/Acinetobacter haemolyticus	P. fluorescens	P. fluorescens	*P. lactis*
BML-PP041	P. putida/*fluorescens*	P. putida	P. putida	P. putida	P. putida
BML-PP042^T^	P. putida/*fluorescens*	P. putida	P. putida	P. putida	*P. urethralis* sp. nov.
BML-PP043	P. putida/*fluorescens*	P. aeruginosa	*P. nitroreducens*	P. aeruginosa	*P. pseudonitroreducens* sp. nov.
BML-PP044	P. putida/*fluorescens*	P. fluorescens	*P. mosselii*	P. putida	*P. mosselii*
BML-PP045	P. putida/*fluorescens*	P. aeruginosa/P. fluorescens/P. mendocina	Pseudomonas *sp.*	P. alcaligenes	*P. tohonis*
BML-PP046	P. putida/*fluorescens*	Acinetobacter haemolyticus	*P. fulva*	P. putida	*P. fulva*
BML-PP047	P. putida/*fluorescens*	P. putida	*P. monteilii*	P. putida	*P. juntendi*
BML-PP048^T^	P. putida/*fluorescens*	P. fluorescens	P. putida	P. putida	*P. faucium* sp. nov.
BML-PP049	P. putida/*fluorescens*	P. aeruginosa/P. fluorescens	P. putida	P. putida	*P. faucium* sp. nov.
BML-PP050	P. putida/*fluorescens*	P. aeruginosa/P. fluorescens	Pseudomonas *sp.*	P. alcaligenes	*P. tohonis*
BML-PP051	P. putida/*fluorescens*	P. putida	*P. monteilii*	P. putida	*P. juntendi*
BML-PP052	P. putida/*fluorescens*	P. aeruginosa/P. fluorescens	*P. mosselii*	P. putida	*P. mosselii*

a Bacteria identification results by ANI/dDDH analysis and automated systems. Agreements with ANI/dDDH analysis are shown in gray.

### Identification based on WGS.

ANI and/or dDDH analysis identified 30 of the 42 isolates as known Pseudomonas species (Table S3). WGS, however, was unable to identify the remaining 12 (Table S3). Comparisons of these 12 isolates with each other by ANI and dDDH analysis showed they belonged to one of 9 different species, respectively (Table S4). Based on morphologic, physiologic, and biochemical properties, we propose that the novel species be named *P. sputi* sp. nov. (BML-PP014^T^), *P. pseudonitroreducens* sp. nov. (BML-PP015^T^, BML-PP034, and BML-PP043), *P. parasichuanensis* sp. nov. (BML-PP020^T^), *P. paraglycinae* sp. nov. (BML-PP023^T^), *P. ceruminis* sp. nov. (BML-PP028^T^), *P. parakoreensis* sp. nov. (BML-PP030^T^), *P. pharyngis* sp. nov. (BML-PP036^T^), *P. urethralis* sp. nov. (BML-PP042^T^), and *P. faucium* sp. nov. (BML-PP048^T^ and BML-PP049).

### Bacterial identification systems compared with ANI and dDDH analysis.

Of the 42 isolates, all four identification systems identified BML-PP041 as P. putida (Table 1). Microscan Walkaway, Vitek 2, and Vitek MS correctly identified BML-PP041, but incorrectly identified or did not identify the other 41 isolates (Table 1). MALDI Biotyper correctly identified 10 isolates but incorrectly identified or did not identify 32 isolates (Table 1).

### Phylogenetic analysis.

**FIG 1:**
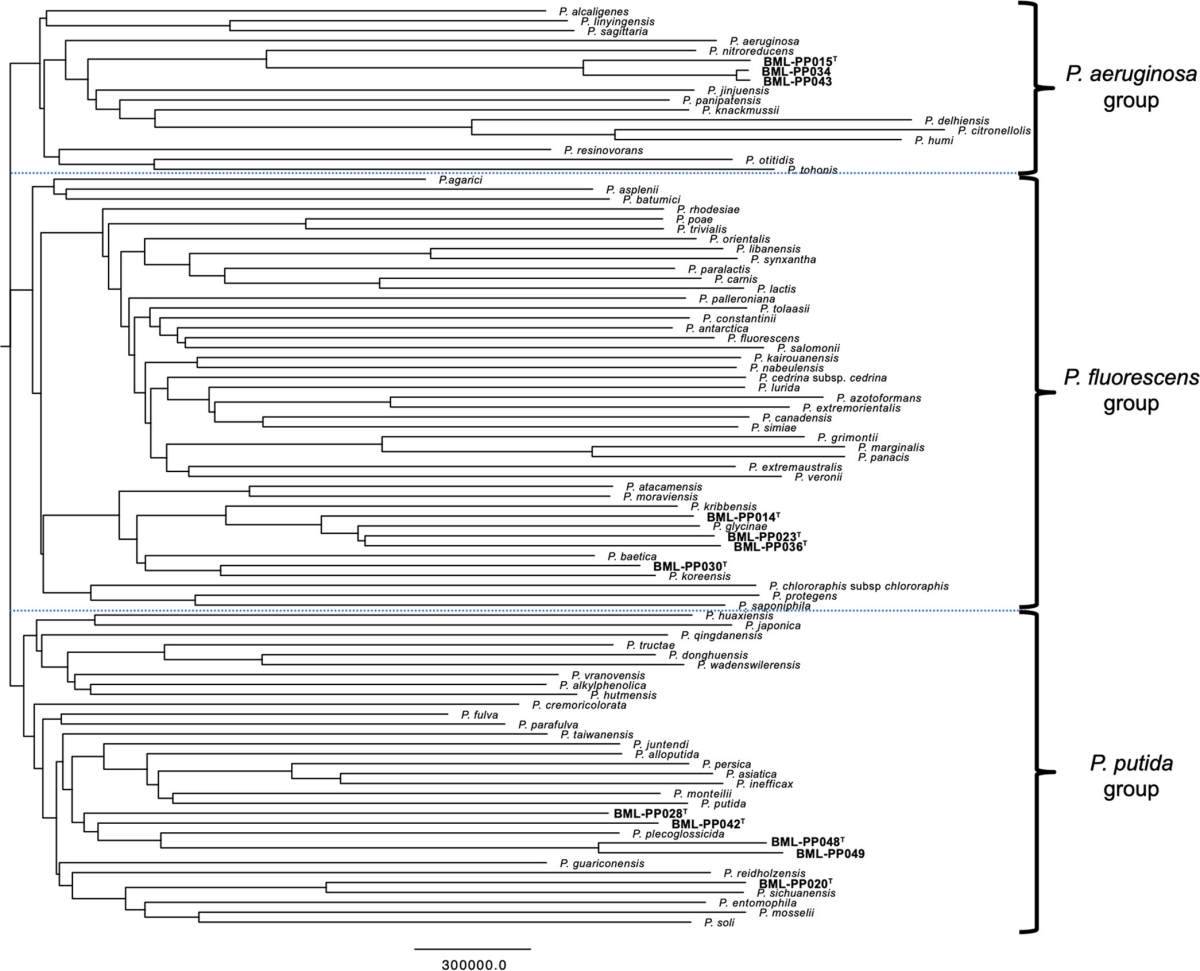
Whole-genome sequence tree for the 12 strains of 9 novel species and the related type strains of Pseudomonas species. A neighbor-joining phylogenetic tree was constructed using pan-genome 18,432,899 single-nucleotide polymorphisms from genomes of the 12 isolates, which were classified into 9 novel species, and the other 81 type strains belonging to the P. aeruginosa, P. fluorescens, and P. putida groups. Scale bar indicates number of nucleotide substitutions. Accession numbers for each sequence are listed in Tables S1 and S2.

Of 12 isolates belonging to the novel Pseudomonas species, 3 belonged to the P. aeruginosa group, 4 to the P. fluorescens group, and 5 to the P. putida group (Fig. 1).

### Biochemical and fatty acid properties of the novel type strains.

Morphologic, physiologic, and biochemical properties of the 9 novel type strains are listed in Table S5 in the supplemental material, with descriptions in the supplemental manuscript. The major fatty acids were C_16:0_ (22.5 to 42.3%), summed feature 3 (C_16:1_*ω7c*/C_16:1_*ω6c*; 12.0 to 40.3%), and summed feature 8 (C_18:1_*ω7c*/C_18:1_*ω6c*; 7.9 to 26.0%) (Table S6).

### MALDI-TOF MS analysis.

**FIG 2:**
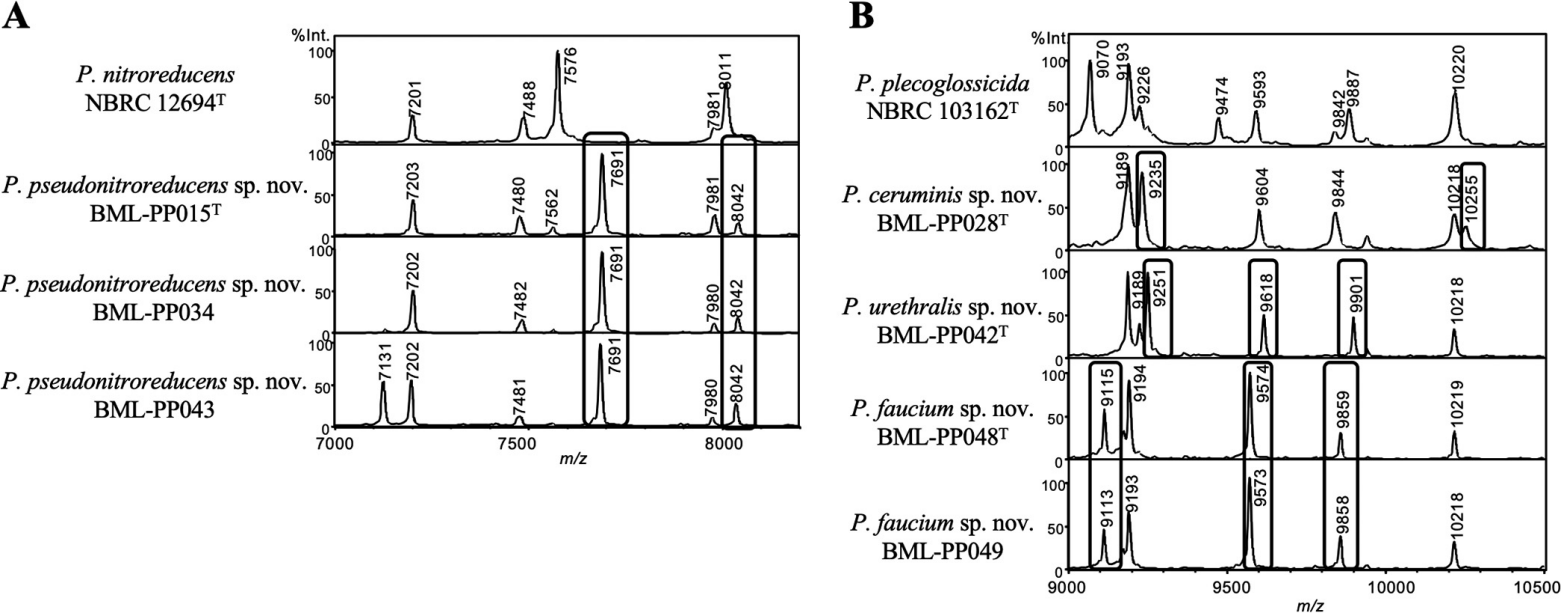
Comparative MALDI-TOF MS profiles of types trains of novel species and related Pseudomonas species. (A) MALDI-TOF MS profiles (7,000 to 8,200 *m/z*) of *P. nitroreducens* NBRC 12694^T^, *P. pseudonitroreducens* sp. nov. BML-PP015^T^, *P. pseudonitroreducens* sp. nov. BML-PP034, and *P. pseudonitroreducens* sp. nov. BML-PP043. *P. nitroreducens* NBRC 12694^T^ had five major peaks at 7,201, 7,488, 7,576, 7,981, and 8,011 *m/z*; whereas *P. pseudonitroreducens* sp. nov. BML-PP015^T^ had six major peaks, three of which, at 7,562, 7,691 and 8,042 *m/z*, differed from those of *P. nitroreducens.* Three strains of *P. pseudonitroreducens* sp. nov. had the same peaks at 7,203, 7,480, 7,691, 7,981 and 8,042 *m/z*. (B) MALDI-TOF MS profiles (9,000 to 10,500 *m/z*) of P. plecoglossicida NBRC 103162^T^, *P. ceruminis* sp. nov. BML-PP028^T^, *P. urethralis* sp. nov. BML-PP042^T^, *P. faucium* sp. nov. BML-PP048^T^, and *P. faucium* sp. nov. BML-PP049. Compared with P. plecoglossicida NBRC 103162^T^, three novel species had unique peaks: at 9,235 and 10,255 *m/z* for *P. ceruminis* sp. nov. (BML-PP028^T^); 9,251, 9,618, and 9,901 *m/z* for *P. urethralis* sp. nov. (BML-PP042^T^), and 9,115, 9,574, and 9,859 *m/z* for *P. faucium* (BML-PP048^T^). The major peaks of one strain of *P. faucium* sp. nov. (BML-PP049) and its type strain, BML-PP048^T^, were almost identical to each other.

The MALDI-TOF MS profiles of the 12 isolates belonging to novel species were compared with profiles of known type strains close to the novel type strains, based on a phylogenetic tree (Fig. 1). The profiles of these novel type strains differed from those of known type strains close to them, whereas the profiles of isolates belonging to the same novel species were almost identical to each other (Fig. 2 and Fig. S1). Compared with the type strain *P. nitroreducens*, the three strains of *P. pseudonitroreducens* sp. nov. had two specific peaks at 7,691 and 8,042 *m/z* (Fig. 2A). Compared with the type strain P. plecoglossicida, the three novel species had unique peaks: at 9,235 and 10,255 *m/z* for *P. ceruminis* sp. nov. (BML-PP028^T^); 9,251, 9,618, and 9,901 *m/z* for *P. urethralis* sp. nov. (BML-PP042^T^); and 9,115, 9,574, and 9,859 *m/z* for *P. faucium* (BML-PP048^T^ and BML-PP049) (Fig. 2B). MALDI-TOF MS detected three unique major peaks for *P. paraglycinae* sp. nov., four for *P. parakoreensis* sp. nov., four for *P. parasichuanensis* sp. nov., two for *P. pharyngis* sp. nov., and one for *P. sputi* sp. nov. (Fig. S1).

### Drug susceptibility testing and drug-resistance genes.

As shown by colistin susceptibility in Table 2, three strains of P. protegens, including the type strain, were highly resistant to colistin, with MICs of 512 to 4,096 μg/mL; and six strains of *P. carnis*, including the type strain, and a *P. lactis* isolate were moderately resistant to colistin, with MICs of 8 to 64 μg/mL. The remaining isolates were susceptible to colistin (Table 2). P. protegens and P. carnis, for which the type strains had been isolated from soil and pork, respectively (18, 19), may be intrinsically resistant to colistin. Most of the 42 isolates were sensitive to other antimicrobial agents, except for aztreonam (Table S7).

**TABLE 2:** Colistin susceptibility of the 42 clinical isolates and two type strains

Isolate	Species	MIC (μg/mL)
Colistin-highly-resistant isolates		
BML-PP019	P. protegens	4,096
BML-PP025	P. protegens	1,024
P. protegens type strain	P. protegens	512

Colistin-resistant isolates		
BML-PP010	P. carnis	64
BML-PP012	P. carnis	32
BML-PP016	P. carnis	32
BML-PP035	P. carnis	32
BML-PP038	P. carnis	32
* P. carnis* type strain	P. carnis	64
BML-PP040	P. lactis	8

Colistin-susceptible isolates		
BML-PP011	P. rhodesiae	0.5
BML-PP013	P. qingdaonensis	0.5
BML-PP014^T^	P. sputi sp. nov.	0.5
BML-PP015^T^	P. pseudonitroreducens sp. nov.	0.25
BML-PP017	P. atacamensis	0.5
BML-PP018	P. fluva	0.5
BML-PP020	P. parasichuanensis sp. nov.	1
BML-PP021	P. juntendi	0.5
BML-PP022	P. asiatica	0.5
BML-PP023^T^	P. paraglycinae sp. nov.	0.5
BML-PP024	P. glycinae	0.5
BML-PP026	P. juntendi	0.5
BML-PP027	P. qingdaonensis	0.25
BML-PP028^T^	P. ceruminis sp. nov.	0.5
BML-PP029	P. otitidis	0.5
BML-PP030^T^	P. parakoreensis sp. nov.	0.5
BML-PP031	P. rhodesiae	1
BML-PP033	P. otitidis	1
BML-PP034	P. pseudonitroreducens sp. nov.	1
BML-PP036^T^	P. pharyngis sp. nov.	0.5
BML-PP037	P. tohonis	0.5
BML-PP039	P. glycinae	0.5
BML-PP041	P. putida	1
BML-PP042^T^	P. urethralis sp. nov.	1
BML-PP043	P. pseudonitroreducens sp. nov.	0.5
BML-PP044	P. mosselii	1
BML-PP045	P. tohonis	0.25
BML-PP046	P. fulva	0.25
BML-PP047	P. juntendi	0.5
BML-PP048^T^	P. faucium sp. nov.	0.5
BML-PP049	P. faucium sp. nov.	0.5
BML-PP050	P. tohonis	0.25
BML-PP051	P. juntendi	0.5
BML-PP052	P. mosselii	1

Assessments of other drug-resistance genes harbored by these 42 isolates showed that 3 of them (BML-PP029, BML-PP030^T^, and BML-PP033) harbored known acquired drug-resistance genes, whereas the remaining 39 isolates did not. Two P. otitidis isolates (BML-PP029 and BML-PP033) harbored *bla*_POM-1_-like genes with 98.3 to 98.8% identity, and one *P. parakoreensis* sp. nov. isolate (BML-PP030^T^) harbored *aadA6* (Table S8).

## DISCUSSION

Improvements are required in automated bacterial identification systems for clinical isolates of species of Pseudomonas, especially isolates belonging to the P. putida and P. fluorescens groups. This study demonstrated that these automated systems performed poorly for identifying isolates belonging to these groups. Specifically, of the 264 P. aeruginosa strains deposited in GenBank, 259 (98%) were correctly identified as P. aeruginosa, whereas all 28 strains deposited as P. fluorescens and all 35 deposited as P. putida had been incorrectly identified (14). In addition, two clinical isolates (BML-PP029 and BML-PP033) of P. otitidis were incorrectly identified or not identified by the automated systems (Table 2), although P. otitidis is a clinically important species belonging to the P. aeruginosa group. These results indicate that Pseudomonas bacteria should be identified using ANI and dDDH analyses.

At present, these WGS-based identification analyses cannot be adapted for use in clinical laboratories. Bacterial identification systems using MALDI-TOF MS are required to correctly identify clinical isolates of Pseudomonas species (17). The strains belonging to the nine novel species had unique major MALDI-TOF MS peaks compared with the type strains of closely related species. Our results suggest that MALDI-TOF MS analysis is able to identify Pseudomonas species; nevertheless, they have 16S rRNA sequences with ≥97% similarity to each other.

Our findings strongly suggest the necessity of using up-to-date databases of bacterial species, especially Pseudomonas species, in automated bacterial identification systems. If bacterial strains collected by individual researchers are used in comparisons, their whole genome sequences should be determined, and the species identified using ANI and dDDH.

Some Pseudomonas species are likely intrinsically resistant to colistin/polymyxin. For example, all strains of P. protegens and *P. carnis* tested in this study, including seven clinical isolates and the two type strains, were resistant to colistin. Other clinically important species known to be intrinsically resistant to colistin/polymyxin include *Burkholderia* spp., Proteus mirabilis, Serratia marcescens, and *Yersinia* spp (20). Epidemiological and bacteriological studies are needed to clarify whether these Pseudomonas spp. have intrinsic resistant to colistin/polymyxin.

This study has some limitations, including the following: (i) the quantity of tested isolates may have been too small to obtain reliable species-specific peaks of MALDI-TOF MS, (ii) a lack of clinical information about the isolates limits estimations of the species’ clinical significance, and (iii) besides P. fluorescens and P. putida, it is necessary to clarify whether other Pseudomonas species besides P. aeruginosa may be isolated from human samples or associated with pathogenesis in humans.

### Conclusion.

Of 42 isolates previously identified as P. putida or P. fluorescens by a bacterial identification system, only 1 was identified as P. putida by four automated identification systems. The 42 isolates included 9 novel Pseudomonas species, which we proposed here. This study indicates that WGS may be the most reliable method for identifying Pseudomonas species, and that MALDI-TOF MS analysis has the potential to complement WGS to reliably identify novel species. However, even up-to-date databases must be treated with caution since there will always be some lag between discovery and valid documentation of novel species.

## MATERIALS AND METHODS

### Bacterial identification using automated systems.

**FIG 3:**
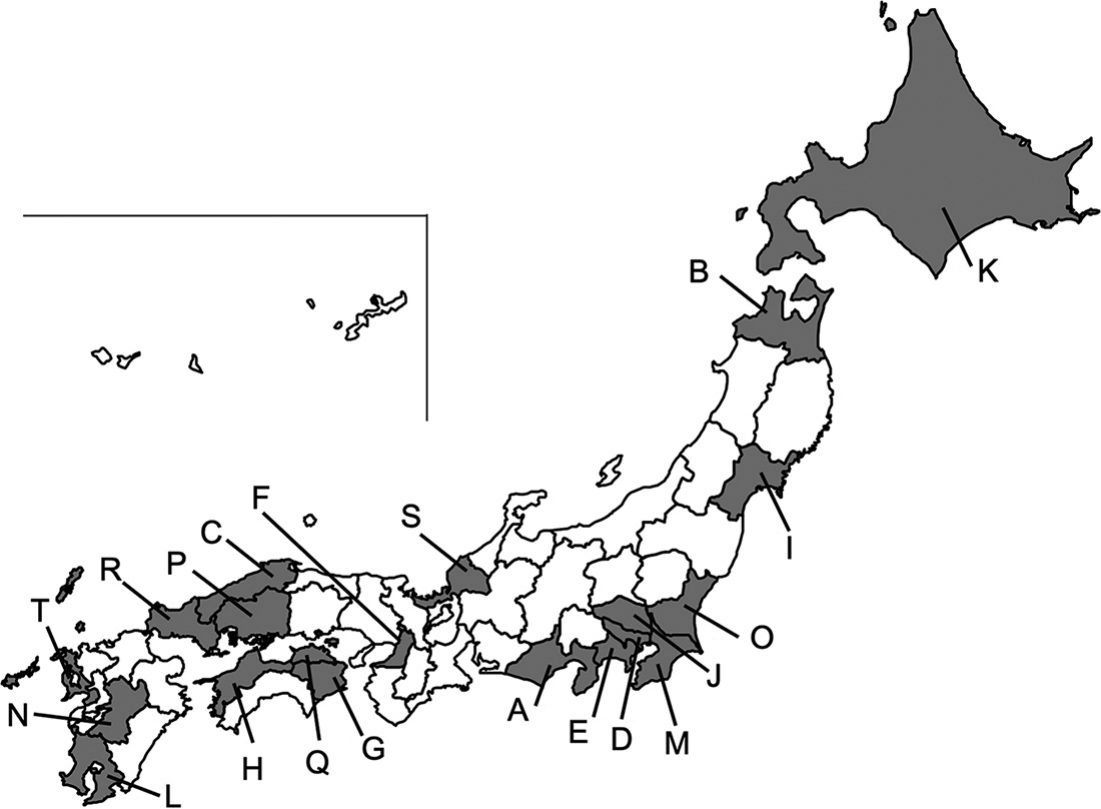
Geographic distribution of the 42 clinical isolates in Japan. The 42 isolates named in Table 3 came from the 20 prefectures shown in the map.

We re-identified 42 isolates, previously identified as P. putida*/fluorescens* by the MicroScan WalkAway system (see Table 3 and Fig. 3 for source details), using the MALDI Biotyper, Vitek 2, and Vitek MS identification systems.

**TABLE 3:** Information about the 42 clinical isolates analyzed in this study

Isolate	Specimen source	Prefecture	Location in Fig. 3
BML-PP010	Sputum	Shizuoka	A
BML-PP011	Sputum	Aomori	B
BML-PP012	Sputum	Shimane	C
BML-PP013	Sputum	Tokyo	D
BML-PP014^T^	Sputum	Kanagawa	E
BML-PP015^T^	Sputum	Osaka	F
BML-PP016	Throat swab	Tokushima	G
BML-PP017	Eye discharge	Tokyo	D
BML-PP018	Urine	Ehime	H
BML-PP019	Catheter urine	Miyagi	I
BML-PP020^T^	Vaginal discharge	Saitama	J
BML-PP021	Sputum	Hokkaido	K
BML-PP022	Sputum	Kanagawa	E
BML-PP023^T^	Sputum	Kanagawa	E
BML-PP024	Sputum	Tokyo	D
BML-PP025	Sputum	Kagoshima	L
BML-PP026	Sputum	Saitama	J
BML-PP027	Sputum	Kanagawa	E
BML-PP028^T^	Ear discharge	Kanagawa	E
BML-PP029	Sputum	Chiba	M
BML-PP030^T^	Throat swab	Kumamoto	N
BML-PP031	Sputum	Shizuoka	A
BML-PP033	Ear discharge	Ibaraki	O
BML-PP034	Throat swab	Hiroshima	P
BML-PP035	Nasopharynx swab	Saitama	J
BML-PP036^T^	Throat swab	Saitama	J
BML-PP037	Sputum	Kagoshima	L
BML-PP038	Sputum	Kagoshima	L
BML-PP039	Sputum	Kagawa	Q
BML-PP040	Sputum	Yamaguchi	R
BML-PP041	Sputum	Fukui	S
BML-PP042^T^	Urethral discharge	Chiba	M
BML-PP043	Throat swab	Saitama	J
BML-PP044	Sputum	Tokushima	G
BML-PP045	Endotracheal sputum	Osaka	F
BML-PP046	Endotracheal sputum	Kanagawa	E
BML-PP047	Urine	Kanagawa	E
BML-PP048^T^	Throat swab	Saitama	J
BML-PP049	Nasal swab	Nagasaki	T
BML-PP050	Sputum	Ehime	H
BML-PP051	Throat swab	Saitama	J
BML-PP052	Pus	Kagoshima	L

### DNA extraction and WGS.

Genomic DNA was extracted using DNeasy Blood and Tissue kits (Qiagen, Hilden, Germany). Genomic libraries were prepared using Nextera XT DNA kits (Illumina, San Diego, CA). Paired-end sequencing was performed using MiSeq Reagent Kits v3 (600-cycle). The sequence reads were quality-trimmed using CLC Genomics Workbench v11 (Qiagen) with the following parameters: quality limit = 0.05, number of 5′-terminal nucleotides to remove = 10, number of 5′-terminal nucleotides to remove = 15, and discarded reads below length = 50, and assembly of the trimmed reads was performed using shovill v1.1.0 with default settings.

### Species identification based on whole genome sequences.

The 42 isolates were re-identified using ANI and dDDH by comparing their whole genome sequences with those of type strains belonging to genus Pseudomonas. ANI and dDDH values were determined by the OrthoANIu algorithm (21) and the Genome to Genome Distance Calculator (GGDC) v2.1 (http://ggdc.dsmz.de/ggdc.php [16]), respectively. In accordance with the International Journal of Systematic and Evolutionary Microbiology (22), the cutoff values of ANI and dDDH between each isolate and the type strain belonging to a species were defined as 95% and 70%, respectively. Isolates not identified by ANI and dDDH were re-analyzed using the Type (Strain) Genome Server (TYGS) (https://tygs.dsmz.de/).

### Drug susceptibility testing.

MICs of drugs against the 42 isolates were determined by microdilution method and interpreted according to CLSI guidelines (M100-S25) (23). Antimicrobial agents were 2-fold diluted in Mueller-Hinton broth (Becton Dickinson, Sparks, MD) at concentrations ranging from 0.0078 to 16 μg/mL for ciprofloxacin and levofloxacin, 0.25 to 4,096 μg/mL for colistin, and 0.25 to 512 μg/mL for the others.

### Drug-resistance genes.

Assembled genome sequences were searched for genes associated with drug resistance using ResFinder v4.1 (https://cge.food.dtu.dk/services/ResFinder/) (24).

### Phylogenetic analysis.

Phylogenetic analysis was performed using kSNP3 v3.1 software, with a k-mer length of 31 (25). A neighbor-joining phylogenetic tree was estimated based on pan-genome 18,432,899 single-nucleotide polymorphisms from genomes, which included recombinant sites, of the 12 isolates classified as new species and the 81 type strains of Pseudomonas. The accession numbers for these genome data are listed in Tables S1 and S2.

### MALDI-TOF MS analysis.

Whole bacterial proteins were analyzed using MALDI-TOF MS, as described previously (26). Cell lysates were mixed with a sinapinic acid matrix solution. MALDI mass spectra were acquired in the range of 2,000 to 30,000 *m/z* in positive-ion linear mode by averaging 1,000 laser shots using an AXIMA Performance (Shimadzu/Kratos, UK) equipped with a pulsed N_2_ laser (λ = 337 nm). Mass calibration was performed using adrenocorticotropic hormone 18 to 39 ([M + H]^+^, 2,466.7 *m/z*) and myoglobin ([M + H]^+^, 16,952.6 *m/z*; [M + 2H]^2+^, 8,476.8 *m/z*) as marker proteins of external calibration.

### Biochemical properties and fatty acids contents of new species.

Biochemical tests were performed using API 20NE (bio Mérieux), API ZYM kits (bio Mérieux) and GN3 MicroPlates (Biolog, Hayward, CA), according to the manufacturers’ instructions. The morphology and dimensions of cells grown for 24 h at 30°C on lysogeny broth (LB) agar (Becton, Dickinson and Co., Franklin Lakes, NJ) were determined by scanning electron microscopy (S4800, Hitachi, Tokyo, Japan). Gram staining was performed as described (26). Fluorescent pigments were detected with King’s A and B agar (Eiken Chemical Co., Ltd., Tokyo, Japan). Physiological tests, including growth at different temperatures (4°C to 44°C at intervals of 4.0°C), pH (pH of 5 to 10, at intervals of 0.5 pH), and NaCl concentrations (1% to 10% [wt/vol], at intervals of 1%) were performed in LB (Becton Dickinson), as described (26). Catalase and oxidase activities were determined using 3% (vol/vol) hydrogen peroxide and Kovács’ reagent, respectively. Fatty acids contents of isolates were analyzed using the Sherlock Microbial Identification (MIDI) system (v6.0) as described (26). Bacterial strains were cultured on tryptic soy broth agar (30 g · L^−1^ tryptic soy broth, 15 g · L^−1^ agar; Becton Dickinson) for 1 day at 30°C, a culture condition frequently used to analyze fatty acids in Pseudomonas novel species (26, 27).

### Data availability.

Whole-genome sequencing data of the 42 isolates were deposited in the GenBank/EMBL/DDBJ database under the accession numbers BQHE00000000 to BQHZ00000000 and BQIA00000000 to BQIT00000000 (Table S1).
