# Correction for Tohya et al., “Whole-Genome Sequencing-Based Re-Identification of Pseudomonas putida/*fluorescens* Clinical Isolates Identified by Biochemical Bacterial Identification Systems”

**DOI:** 10.1128/spectrum.04305-22

**Published:** 2023-02-21

**Authors:** Mari Tohya, Kanae Teramoto, Shin Watanabe, Tomomi Hishinuma, Masahito Shimojima, Miho Ogawa, Tatsuya Tada, Yoko Tabe, Teruo Kirikae

## AUTHOR CORRECTION

Volume 10, no. 2, e02491-21, 2022, https://doi.org/10.1128/spectrum.02491-21. The following taxonomy section should appear in the main article and not in the supplemental material. (A revised supplemental material file is included with this Author Correction.)

## DESCRIPTION OF NINE NOVEL SPECIES

### Description of Pseudomonas *sputi* sp. nov.

Pseudomonas sputi (spu'ti. L. gen. n. sputi of sputum).

The cells of this species are aerobic, Gram-negative, non-spore-forming, motile, rod-shaped cells, measuring 1.0 to 2.0 μm in length and 0.5 to 0.7 μm in width. Colonies on Luria broth agar were circular, convex in shape with a creamy color, and usually 1 to 3 mm in diameter after growth for 2 days at 30°C. These bacteria grew at temperatures between 4 and 36°C, at pH 6.0 to 9.0, and in the presence of 0 to 6.0% (wt/vol) NaCl. They produced catalase and cytochrome oxidase, as well as fluorescent pigments, on King’s B agar at 30°C. In API 20NE tests, BML-PP014^T^ was positive for l-arginine, gelatin, glucose, l-arabinose, d-mannose, d-mannitol, *N*-acetyl-d-glucosamine, potassium glucose, *n*-capric acid, dl-malic acid, and sodium citrate. In API ZYM tests, this strain was positive for alkaline phosphatase, esterase (C_4_), esterase lipase (C_8_), leucine arylamidase, valine arylamidase, acid phosphatase, and naphthol-AS-BI-phosphohydrolase activities. In Biolog GN3 tests, this strain was positive for *N*-acetyl-d-glucosamine, α-d-glucose, d-mannose, d-fructose, d-galactose, d-fucose, l-fucose, inosine, d-mannitol, d-arabitol, glycerol, d-fructose-6-PO_4_, l-alanine, l-arginine, l-aspartic acid, l-glutamic acid, l-pyroglutamic acid, l-serine, d-galacturonic acid, l-galactonic acid lactone, d-gluconic acid, d-glucuronic acid, glucuronamide, mucic acid, quinic acid, d-saccharic acid, methyl pyruvate, l-lactic acid, citric acid, α-keto-glutaric acid, l-malic acid, γ-amino-butyric acid, β-hydroxy-d,l-butyric acid, propionic acid, and acetic acid. The major fatty acids were summed feature 3 (C_16:1ω7c_/C_16:1ω6c_; 37.5%), C_16:0_ (28.8%), and summed feature 8 (C_18:1ω7c_/C_18:1ω6c_; 9.9%). The type strain is BML-PP014^T^ (= JCM 34577^T^, = LMG 32246^T^), isolated from a sputum sample of a patient in Japan. The G+C content of the type strain is 60.30 mol%.

### Description of Pseudomonas *pseudonitroreducens* sp. nov.

Pseudomonas pseudonitroreducens [pseudês, Gr. masc./fem. adj., false; nitro.re.du'cens. L. n. nitrum nitrate; L. part. adj. reducens converting to a different state; N. L. adj. nitroreducens reducing nitrate; pseudonitroreducens, N.L. masc. adj., a false (Pseudomonas) *nitroreducens*].

The cells of this species are aerobic, Gram-negative, non-spore-forming, motile, rod-shaped cells, being 1.0 to 2.0 μm in length and 0.5 to 0.7 μm in width. Colonies on Luria broth agar were circular, convex in shape with a creamy color, and usually 1 to 1.5 mm in diameter after growth for 2 days at 30°C. These bacteria grew at temperatures between 8 and 36°C, at pH 5.5 to 9.0, and in the presence of 0 to 6.0% (wt/vol) NaCl. They produced catalase and cytochrome oxidase, as well as fluorescent pigments, on King’s B agar at 30°C. In API 20NE tests, BML-PP015^T^ was positive for potassium nitrate, l-arginine, glucose, potassium glucose, *n*-capric acid, adipic acid, dl-malic acid, sodium citrate, and phenylmercuric acetate. In API ZYM tests, this strain was positive for alkaline phosphatase, esterase (C_4_), esterase lipase (C_8_), leucine arylamidase, valine arylamidase, acid phosphatase, and naphthol-AS-BI-phosphohydrolase activities. In Biolog GN3 tests, this strain was positive for α-d-glucose, d-fucose, l-fucose, glycerol, d-fructose-6-PO_4_, l-alanine, l-arginine, l-aspartic acid, l-glutamic acid, l-serine, l-galactonic acid lactone, d-gluconic acid, glucuronamide, quinic acid, *p*-hydroxy-phenylacetic acid, methyl pyruvate, l-lactic acid, citric acid, α-keto-glutaric acid, l-malic acid, γ-amino-butyric acid, β-hydroxy-d,l-butyric acid, propionic acid, and acetic acid. The major fatty acids are summed feature 3 (C_16:1ω7c_/C_16:1ω6c_; 27.4%), summed feature 8 (C_18:1ω7c_/C_18:1ω6c_; 26.0%), and C_16:0_ (22.5%). The type strain is BML-PP015^T^ (= JCM 34578^T^, = LMG 32247^T^), isolated from a sputum sample of a patient in Japan. The G+C content of the type strain is 64.50 mol%.

### Description of Pseudomonas *parasichuanensis* sp. nov.

Pseudomonas parasichuanensis [pa.ra. Gr. prep. beside, alongside, near, like; si.chuan.en'sis. N.L. fem. adj. referring to Sichuan Province, People’s Republic of China and the specific epithet of a Pseudomonas species; parasichuanensis. N.L. masc. adj. next to (Pseudomonas) *sichuanensis*].

The cells of this species are aerobic, Gram-negative, non-spore-forming, motile, rod-shaped cells, being 1.0 to 1.5 μm in length and 0.6 to 0.9 μm in width. Colonies on Luria broth agar were circular, convex in shape with a creamy color, and usually 1 to 2 mm in diameter after growth for 2 days at 30°C. These bacteria grew at temperatures between 8 and 36°C, at pH 6.0 to 9.0, and in the presence of 0 to 6.0% (wt/vol) NaCl. They produced catalase and cytochrome oxidase, as well as fluorescent pigments, on King’s B agar at 30°C. In API 20NE tests, BML-PP020^T^ was positive for l-arginine, gelatin, glucose, potassium gluconate, *n*-capric acid, dl-malic acid, sodium citrate, and phenylmercuric acetate. In API ZYM tests, the strain was positive for alkaline phosphatase, esterase (C_4_), esterase lipase (C_8_), leucine arylamidase, valine arylamidase, trypsin, acid phosphatase, naphthol-AS-BI-phosphohydrolase, and β-galactosidase activities. In Biolog GN3 tests, this strain was positive for gentiobiose, α-d-glucose, d-mannose, d-fructose, d-galactose, 3-methyl glucose, d-fucose, l-fucose, l-rhamnose, glycerol, d-fructose-6-PO_4_, d-aspartic acid, d-serine, l-alanine, l-arginine, l-aspartic acid, l-glutamic acid, l-histidine, l-pyroglutamic acid, l-serine, d-galacturonic acid, l-galactonic acid lactone, d-gluconic acid, d-glucuronic acid, glucuronamide, mucic acid, quinic acid, d-saccharic acid, *p*-hydroxy-phenylacetic acid, methyl pyruvate, l-lactic acid, citric acid, α-keto-glutaric acid, d-malic acid, l-malic acid, Tween 40, γ-amino-butyric acid, α-hydroxy-butyric acid, β-hydroxy-d,l-butyric acid, α-keto-butyric acid, acetoacetic acid, propionic acid, acetic acid, and formic acid. The major fatty acids are summed feature 3 (C_16:1ω7c_/C_16:1ω6c_; 32.6%), C_16:0_ (27.7%), and summed feature 8 (C_18:1ω7c_/C_18:1ω6c_; 15.0%). The type strain is BML-PP020^T^ (= JCM 34580^T^, = LMG 32249^T^), isolated from a vaginal discharge sample of a patient in Japan. The G+C content of the type strain is 64.19 mol%.

### Description of Pseudomonas *paraglycinae* sp. nov.

Pseudomonas paraglycinae [pa.ra. Gr. prep. beside, alongside, near, like; gly.ci'nae. N.L. gen. n. glycinae of Glycine max, soybean and the specific epithet of a Pseudomonas species; paraglycinae. N.L. masc. adj. next to (Pseudomonas) *glycinae*].

The cells of this species are aerobic, Gram-negative, non-spore-forming, motile, rod-shaped cells, being 1.0 to 2.0 μm in length and 0.6 to 0.8 μm in width. Colonies on Luria broth agar were circular, convex in shape with a creamy color, and usually 2 to 4 mm in diameter after growth for 2 days at 30°C. These bacteria grew at temperatures between 4 and 36°C, at pH 6.0 to 9.0, and in the presence of 0 to 6.0% (wt/vol) NaCl. They produced catalase and cytochrome oxidase, as well as fluorescent pigments, on King’s B agar at 30°C. In API 20NE tests, BML-PP023^T^ was positive for l-arginine, gelatin, glucose, l-arabinose, d-mannose, d-mannitol, *N*-acetyl-d-glucosamine, potassium gluconate, *n*-capric acid, dl-malic acid, and sodium citrate. In API ZYM tests, the strain was positive for alkaline phosphatase, esterase (C_4_), esterase lipase (C_8_), lipase (C_14_), leucine arylamidase, valine arylamidase, trypsin, acid phosphatase, and naphthol-AS-BI-phosphohydrolase activities. In Biolog GN3 tests, this strain was positive for *N*-acetyl-d-glucosamine, α-d-glucose, d-mannose, d-fructose, d-galactose, d-fucose, l-fucose, inosine, d-mannitol, d-arabitol, *myo*-inositol, glycerol, d-fructose-6-PO_4_, l-alanine, l-arginine, l-aspartic acid, l-glutamic acid, l-pyroglutamic acid, l-serine, l-galactonic acid lactone, d-gluconic acid, glucuronamide, mucic acid, quinic acid, d-saccharic acid, methyl pyruvate, l-lactic acid, citric acid, α-keto-glutaric acid, l-malic acid, Tween 40, γ-amino-butyric acid, β-hydroxy-d,l-butyric acid, propionic acid, and acetic acid. The major fatty acids are summed feature 3 (C_16:1ω7c_/C_16:1ω6c_; 39.3%), C_16:0_ (31.4%), and summed feature 8 (C_18:1ω7c_/C_18:1ω6c_; 9.9%). The type strain is BML-PP023^T^ (= JCM 34581^T^, = LMG 32250^T^), isolated from a sputum sample of a patient in Japan. The G+C content of the type strain is 60.68 mol%.

### Description of Pseudomonas *ceruminis* sp. nov.

Pseudomonas ceruminis (ceru'mi.nis L. gen. neut.n. ceruminis of cerumen).

The cells of this species are aerobic, Gram-negative, non-spore-forming, motile, rod-shaped cells, being 1.0 to 2.0 μm in length and 0.7 to 0.8 μm in width. Colonies on Luria broth agar were circular, convex in shape with a creamy color, and usually 1 to 2 mm in diameter after growth for 2 days at 30°C. These bacteria grew at temperatures between 8 and 36°C, at pH 6.0 to 9.0, and in the presence of 0 to 6.0% (wt/vol) NaCl. They produced catalase and cytochrome oxidase, as well as fluorescent pigments, on King’s B agar at 30°C. In API 20NE tests, BML-PP028^T^ was positive for l-arginine, glucose, d-mannose, d-mannitol, potassium glucose, *n*-capric acid, dl-malic acid, sodium citrate, and phenylmercuric acetate. In API ZYM tests, the strain was positive for alkaline phosphatase, esterase (C_4_), esterase lipase (C_8_), leucine arylamidase, valine arylamidase, trypsin, acid phosphatase, and naphthol-AS-BI-phosphohydrolase activities. In Biolog GN3 tests, this strain was positive for gentiobiose, d-melibiose, α-d-glucose, d-mannose, d-fructose, d-galactose, 3-methyl glucose, d-fucose, l-fucose, l-rhamnose, inosine, d-mannitol, d-arabitol, glycerol, d-fructose-6-PO_4_, d-serine, l-alanine, l-arginine, l-aspartic acid, l-glutamic acid, l-histidine, l-pyroglutamic acid, l-serine, d-galacturonic acid, l-galactonic acid lactone, d-gluconic acid, d-glucuronic acid, glucuronamide, quinic acid, methyl pyruvate, l-lactic acid, citric acid, α-keto-glutaric acid, d-malic acid, l-malic acid, bromo-succinic acid, γ-amino-butyric acid, α-hydroxy-butyric acid, β-hydroxy-d,l-butyric acid, α-keto-butyric acid, acetoacetic acid, propionic acid, acetic acid, and formic acid. The major fatty acids are C_16:0_ (42.3%), summed feature 3 (C_16:1ω7c_/C_16:1ω6c_; 17.5%), and C_17:0cyclo_ (11.9%). The type strain is BML-PP028^T^ (= JCM 34110^T^, = DSM 111127^T^), isolated from an ear discharge sample of a patient in Japan. The G+C content of the type strain is 61.67 mol%.

### Description of Pseudomonas *parakoreensis* sp. nov.

Pseudomonas parakoreensis [pa.ra. Gr. prep. beside, alongside, near, like; ko.re.en'sis N.L. fem. adj. koreensis, pertaining to Korea and the specific epithet of a Pseudomonas species; parakoreensis. N.L. masc. adj. next to (Pseudomonas) *koreensis*].

The cells of this species are aerobic, Gram-negative, non-spore-forming, motile, rod-shaped cells, being 1.0 to 2.0 μm in length and 0.6 to 0.7 μm in width. Colonies on Luria broth agar were circular, convex in shape with a creamy color, and usually 1 to 3 mm in diameter after growth for 2 days at 30°C. These bacteria grew at temperatures between 4 and 36°C, at pH 6.0 to 8.5, and in the presence of 0 to 5.0% (wt/vol) NaCl. They produced catalase and cytochrome oxidase, as well as fluorescent pigments, on King’s B agar at 30°C. In API 20NE tests, BML-PP030^T^ was positive for l-arginine, gelatin, glucose, l-arabinose, d-mannose, d-mannitol, *N*-acetyl-d-glucosamine, potassium glucose, *n*-capric acid, dl-malic acid, and sodium citrate. In API ZYM tests, the strain was positive for alkaline phosphatase, esterase (C_4_), esterase lipase (C_8_), lipase (C_14_), leucine arylamidase, valine arylamidase, trypsin, acid phosphatase, and naphthol-AS-BI-phosphohydrolase activities. In Biolog GN3 tests, this strain was positive for *N*-acetyl-d-glucosamine, α-d-glucose, d-mannose, d-galactose, d-fucose, l-fucose, inosine, d-mannitol, d-arabitol, glycerol, d-fructose-6-PO_4_, d-serine, l-alanine, l-arginine, l-aspartic acid, l-glutamic acid, l-histidine, l-pyroglutamic acid, l-serine, d-galacturonic acid, l-galactonic acid lactone, d-gluconic acid, d-glucuronic acid, glucuronamide, mucic acid, quinic acid, d-saccharic acid, methyl pyruvate, l-lactic acid, citric acid, α-keto-glutaric acid, l-malic acid, bromo-succinic acid, Tween 40, γ-amino-butyric acid, β-hydroxy-d,l-butyric acid, propionic acid, and acetic acid. The major fatty acids are summed feature 3 (C_16:1ω7c_/C_16:1ω6c_; 37.8%), C_16:0_ (31.6%), and summed feature 8 (C_18:1ω7c_/C_18:1ω6c_; 10.7%). The type strain is BML-PP030^T^ (= JCM 34582^T^, = LMG 32251^T^), isolated from a throat swab sample of a patient in Japan. The G+C content of the type strain is 60.09 mol%.

### Description of Pseudomonas *pharyngis* sp. nov.

Pseudomonas pharyngis (pha.ryn'gis Gr. n. phaynx throat; Gr. Gen. n. pharyngis of the throat).

The cells of this species are aerobic, Gram-negative, non-spore-forming, motile, rod-shaped cells, being 1.0 to 1.5 μm in length and 0.8 to 0.9 μm in width. Colonies on Luria broth agar were circular, convex in shape with a creamy color, and usually 1 to 5 mm in diameter after growth for 2 days at 30°C. These bacteria grew at temperatures between 4 and 36°C, at pH 6.0 to 9.0, and in the presence of 0 to 6.0% (wt/vol) NaCl. They produced catalase and cytochrome oxidase, as well as fluorescent pigments, on King’s B agar at 30°C. In API 20NE tests, BML-PP036^T^ was positive for l-arginine, gelatin, glucose, l-arabinose, d-mannose, d-mannitol, *N*-acetyl-d-glucosamine, potassium glucose, *n*-capric acid, dl-malic acid, and sodium citrate. In API ZYM tests, the strain was positive for alkaline phosphatase, esterase (C_4_), esterase lipase (C_8_), lipase (C_14_), leucine arylamidase, valine arylamidase, trypsin, acid phosphatase, and naphthol-AS-BI-phosphohydrolase activities. In Biolog GN3 tests, this strain was positive for *N-*acetyl-d-glucosamine, α-d-glucose, d-mannose, d-fructose, d-galactose, d-fucose, l-fucose, inosine, d-mannitol, d-arabitol, glycerol, d-fructose-6-PO_4_, d-serine, l-alanine, l-arginine, l-aspartic acid, l-glutamic acid, l-pyroglutamic acid, l-serine, l-galactonic acid lactone, d-gluconic acid, glucuronamide, mucic acid, quinic acid, d-saccharic acid, methyl pyruvate, l-lactic acid, citric acid, α-keto-glutaric acid, l-malic acid, Tween 40, γ-amino-butyric acid, β-hydroxy-d,l-butyric acid, propionic acid, and acetic acid. The major fatty acids are summed feature 3 (C_16:1ω7c_/C_16:1ω6c_; 40.3%), C_16:0_ (31.9%), and summed feature 8 (C_18:1ω7c_/C_18:1ω6c_; 8.9%). The type strain is BML-PP036^T^ (= JCM 34583^T^, = LMG 32252^T^), isolated from a throat swab sample of a patient in Japan. The G+C content of the type strain is 60.42 mol%.

### Description of Pseudomonas *urethralis* sp. nov.

Pseudomonas urethralis (L. fem. n. urethra, the excretory canal of the urine, the urethra; L. fem. suff. -alis, suffix denoting pertaining to; N.L. fem. adj. urethralis, of or pertaining to the urethra).

The cells of this species are aerobic, Gram negative, non-spore-forming, motile, rod-shaped cells, being 1.0 to 2.0 μm in length and 0.8 to 1.0 μm in width. Colonies on Luria broth agar were circular, convex in shape with a creamy color, and usually 1 to 3 mm in diameter after growth for 2 days at 30°C. These bacteria grew at temperatures between 8 and 40°C, at pH 6.0 to 9.0, and in the presence of 0 to 6.0% (wt/vol) NaCl. They produced catalase and cytochrome oxidase, as well as fluorescent pigments, on King’s A and B agar at 30°C. In API 20NE tests, BML-PP042^T^ was positive for l-arginine, glucose, l-arabinose, d-mannose, potassium glucose, *n*-capric acid, dl-malic acid, and sodium citrate. In API ZYM tests, the strain was positive for alkaline phosphatase, esterase (C_4_), esterase lipase (C_8_), leucine arylamidase, valine arylamidase, trypsin, acid phosphatase, and naphthol-AS-BI-phosphohydrolase. In Biolog GN3 tests, this strain was positive for α-d-glucose, d-mannose, d-fructose, d-galactose, d-fucose, l-fucose, inosine, glycerol, d-fructose-6-PO_4_, d-serine, l-alanine, l-arginine, l-aspartic acid, l-glutamic acid, l-histidine, l-pyroglutamic acid, l-serine, d-galacturonic acid, l-galactonic acid lactone, d-gluconic acid, d-glucuronic acid, glucuronamide, mucic acid, quinic acid, d-saccharic acid, methyl pyruvate, l-lactic acid, citric acid, α-keto-glutaric acid, l-malic acid, Tween 40, γ-amino-butyric acid, α-hydroxy-butyric acid, β-hydroxy-d,l-butyric acid, α-keto-butyric acid, acetoacetic acid, propionic acid, acetic acid, and formic acid. The major fatty acids are C_16:0_ (40.0%), summed feature 3 (C_16:1ω7c_/C_16:1ω6c_; 23.4%), and summed feature 8 (C_18:1ω7c_/C_18:1ω6c_; 13.0%). The type strain is BML-PP042^T^ (= JCM 34111^T^, = DSM 111128^T^), isolated from a urethral discharge sample of a patient in Japan. The G+C content of the type strain is 62.71 mol%.

### Description of Pseudomonas *faucium* sp. nov.

Pseudomonas faucium (fau'ci.um L. gen. pl. n. faucium, of the throat).

The cells of this species are aerobic, Gram negative, non-spore-forming, motile, rod-shaped cells, being 1.0 to 2.0 μm in length and 0.6 to 0.8 μm in width, Colonies on Luria broth agar were circular, convex in shape with a creamy color, and usually 1 to 3 mm in diameter after growth for 2 days at 30°C. These bacteria grew at temperatures between 8 and 36°C, at pH 6.0 to 9.0, and in the presence of 0 to 6.0% (wt/vol) NaCl. They produced catalase and cytochrome oxidase, as well as fluorescent pigments, on King’s B agar at 30°C. In API 20NE tests, BML-PP048^T^ was positive for potassium nitrate, l-arginine, glucose, d-mannose, potassium glucose, *n*-capric acid, dl-malic acid, sodium citrate, and phenylmercuric acetate. In API ZYM tests, the strain was positive for alkaline phosphatase, esterase (C_4_), esterase lipase (C_8_), leucine arylamidase, valine arylamidase, acid phosphatase, and naphthol-AS-BI-phosphohydrolase. In Biolog GN3 tests, this strain was positive for α-d-glucose, d-mannose, d-fructose, d-galactose, 3-methyl glucose, d-fucose, l-fucose, l-rhamnose, inosine, glycerol, d-fructose-6-PO_4_, d-serine, l-alanine, l-arginine, l-aspartic acid, l-glutamic acid, l-histidine, l-pyroglutamic acid, l-serine, d-galacturonic acid, l-galactonic acid lactone, d-gluconic acid, d-glucuronic acid, glucuronamide, quinic acid, methyl pyruvate, l-lactic acid, citric acid, α-keto-glutaric acid, d-malic acid, l-malic acid, bromo-succinic acid, γ-amino-butyric acid, α-hydroxy-butyric acid, β-hydroxy-d,l-butyric acid, α-keto-butyric acid, acetoacetic acid, propionic acid, acetic acid, and formic acid. The major fatty acids are C_16:0_ (42.2%), C_17:0cyclo_ (19.1%), and summed feature 3 (C_16:1ω7c_/C_16:1ω6c_; 12.0%). The type strain is BML-PP048^T^ (= JCM 34112^T^, = DSM 111130^T^), isolated from a throat swab sample of a patient in Japan. The G+C content of the type strain is 64.19 mol%.

