# Supplementary material for: Whole-Genome Sequencing-Based Re-Identification of Pseudomonas putida/fluorescens Clinical Isolates Identified by Biochemical Bacterial Identification Systems
Source: Microbiol Spectr. 2022 Apr 7;10(2):e02491-21. doi: 10.1128/spectrum.02491-21 (PMC9045174; doi:10.1128/spectrum.02491-21)
Supplement: SUPPLEMENTAL FILE 1 — Supplemental material. Download SPECTRUM02491-21_Supp_1_seq5.pdf, PDF file, 0.6 MB [file spectrum02491-21_supp_1_seq5.pdf]

Microbiology Spectrum
Supplemental Materials

Whole Genome Sequencing-based Re-identification of *Pseudomonas putida/fluorescens* Clinical Isolates Identified by Biochemical Bacterial Identification Systems

Mari Tohya[1, 2], Kanae Teramoto[3], Shin Watanabe[2], Tomomi Hishinuma[1], Masahito Shimojima[4,5], Miho Ogawa[5], Tatsuya Tada[1], Yoko Tabe[6], Teruo Kirikae[1*]

[1]Department of Microbiology, Juntendo University School of Medicine, Tokyo, Japan, [2]Department of Microbiome Research, Juntendo University School of Medicine, Tokyo, Japan, [3]Koichi Tanaka Mass Spectrometry Research Laboratory, Kyoto, Japan, [4]SUGIYAMA-GEN Co., Ltd., Tokyo, Japan, [5]BML, Inc., Saitama, Japan, [6]Department of Clinical Laboratory Medicine, Juntendo University Graduate School of Medicine, Tokyo, Japan

[*]**Corresponding author:** Teruo Kirikae, M.D., Ph.D., t-kirikae@juntendo.ac.jp

Address: Department of Microbiology, Juntendo University School of Medicine, Tokyo, Japan, 2-1-1 Hongo, Bunkyo-ku, Tokyo 113-8421, Japan

Phone: (81) 3 5802 1041, **Fax:** (81) 3 5684 7830

Running Title: Re-identification of *Pseudomonas* isolates

KEYWORDS: *Pseudomonas*, human pathogen, re-identification

Figure S1. Comparative MALDI-TOF MS profiles of type strains of novel species and related *Pseudomonas* species.

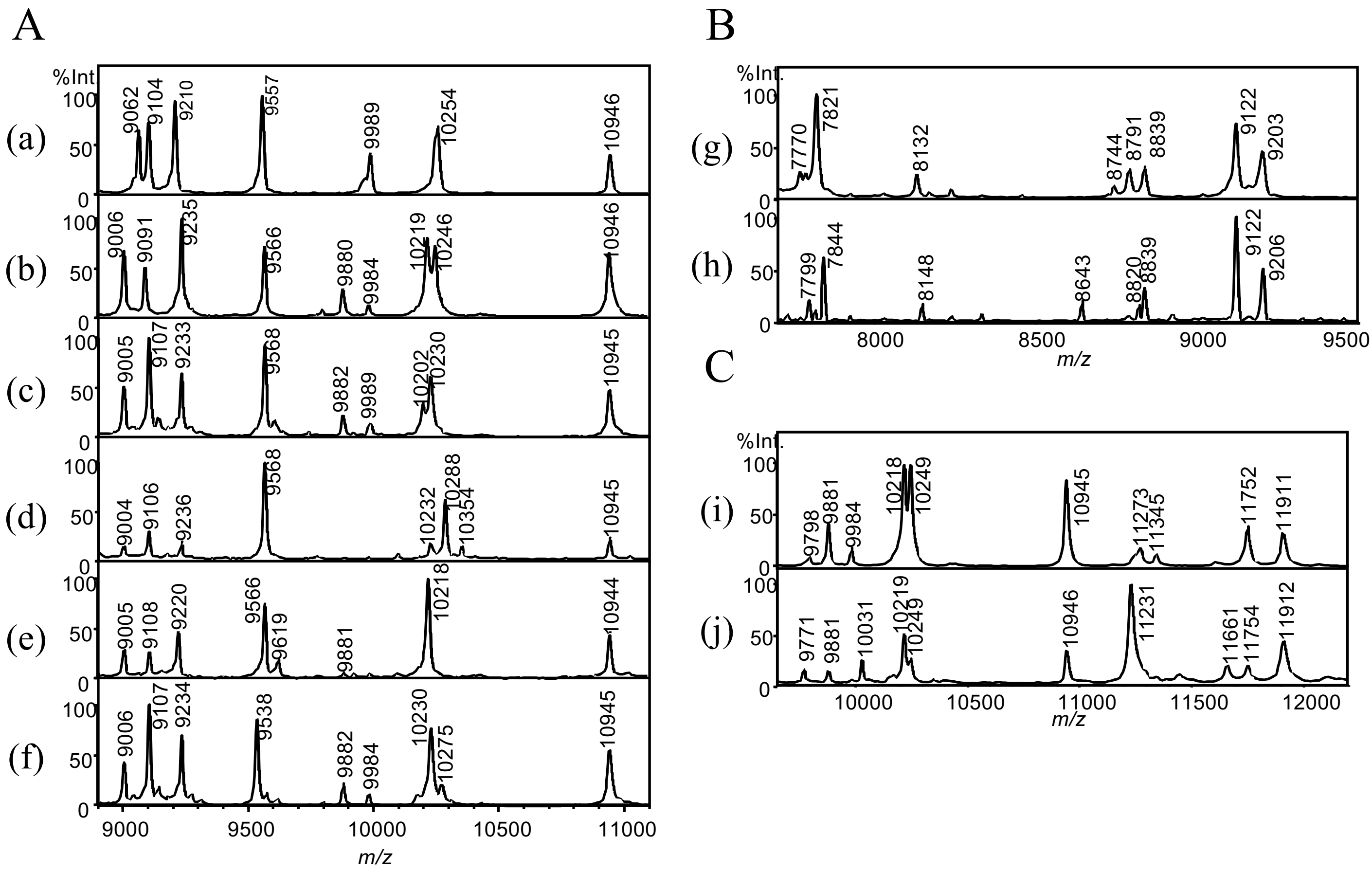

A: MALDI-TOF MS profiles (m/z 9000-11100) of (a) *P. fluorescens* NBRC 14160^T, (b) *P. koreensis* JCM 14769^T, (c) *P. glycinae* LMG 30275^T, (d) *P. sputi* sp. nov. BML-PP014^T, (e) *P. paraglycinae* sp. nov. BML-PP023^T and (f) *P. pharyngis* sp. nov. BML-PP036^T. B: MALDI-TOF MS profiles (m/z 7700-9500) of (g) *P. sichuanensis* JCM 32906^T and (h) *P. parasichuanensis* sp. nov. BML-PP020^T. C: MALDI-TOF MS profiles (m/z 9700-12200) of (i) *P. koreensis* JCM 32906^T and (j) *P. parakoreensis* sp. nov. BML-PP030^T.

MALDI-TOF MS detected one to four unique major peaks in each of the five novel species, including at *m/z* 9220, 9619 and 10218 for *P. paraglycinae* sp. nov.; at *m/z* 9771, 10031, 11231 and 11661 for *P. parakoreensis* sp. nov.; at *m/z* 7799, 7844, 8148 and 8643 for *P. parasichuanensis* sp. nov.; at *m/z* 9538 and 10275 for *P. pharyngis* sp. nov.; and at *m/z* 10354 for *P. sputi* sp. nov.

Table S1. Accession numbers of whole genome sequence data for the clinical 42 isolates

Isolate	Accession No.
BML-PP010	BQHE00000000
BML-PP011	BQHF00000000
BML-PP012	BQHG00000000
BML-PP013	BQHH00000000
BML-PP014[T]	BQHI00000000
BML-PP015[T]	BQHJ00000000
BML-PP016	BQHK00000000
BML-PP017	BQHL00000000
BML-PP018	BQHM00000000
BML-PP019	BQHN00000000
BML-PP020[T]	BQHO00000000
BML-PP021	BQHP00000000
BML-PP022	BQHQ00000000
BML-PP023[T]	BQHR00000000
BML-PP024	BQHS00000000
BML-PP025	BQHT00000000
BML-PP026	BQHU00000000
BML-PP027	BQHV00000000
BML-PP028[T]	BQHW00000000
BML-PP029	BQHX00000000
BML-PP030[T]	BQHY00000000
BML-PP031	BQHZ00000000
BML-PP033	BQIA00000000
BML-PP034	BQIB00000000
BML-PP035	BQIC00000000
BML-PP036[T]	BQID00000000
BML-PP037	BQIE00000000
BML-PP038	BQIF00000000
BML-PP039	BQIG00000000
BML-PP040	BQIH00000000
BML-PP041	BQII00000000
BML-PP042[T]	BQIJ00000000
BML-PP043	BQIK00000000
BML-PP044	BQIL00000000
BML-PP045	BQIM00000000
BML-PP046	BQIN00000000
BML-PP047	BQIO00000000
BML-PP048[T]	BQIP00000000
BML-PP049	BQIQ00000000
BML-PP050	BQIR00000000
BML-PP051	BQIS00000000
BML-PP052	BQIT00000000

Table S2. Accession numbers of whole genome sequence data for type strains

Species	Strains	Accession No.
P. aeruginosa	DSM 50071^T	NZ_CP012001
P. agarici	NCPPB 2289^T	AKBQ01000000
P. alcaligenes	NBRC 14159^T	BATI01000000
P. alkylphenolica	KL28^T	CP009048
P. alloputida	Kh7^T	OLKK01000000
P. antarctica	DSM 15318^T	UYXQ01000000
P. asiatica	RYU5^T	NZ_BLJF01000000
P. asplenii	ATCC 23835^T	NZ_LT629777
P. atacamensis	M7D1^T	SSBS01000000
P. azotoformans	DSM 18862^T	MNPV01000000
P. baetica	a390^T	PKLC01000000
P. batumici	UCM B-321^T	JXDG01000000
P. canadensis	2-92^T	AYTD01000000
P. carnis	B4-1^T	NZ_CABIVL01000000
P. cedrina subsp. *cedrina*	DSM 117516^T	UYXV01000000
P. chlororaphis subsp. *chlororaphis*	DSM 50083^T	CP027712
P. citronellolis	NBRC 103043^T	BCZY01000000
P. constantinii	LMG 22119^T	MDDR01000000
P. cremoricolorata	NBRC 16634^T	AUEA01000000
P. delhiensis	CCM 7361^T	FNEC01000000
P. donghuensis	HYS^T	AJJP01000000
P. entomophila	L48^T	CT573326
P. extremaustralis	DSM 17835^T	LT629689
P. extremorientalis	LMG 19695^T	MDGK01000000
P. fluorescens	NCTC 10038^T	LS483372
P. fulva	NBRC 16637^T	BBIQ01000000
P. glycinae	MS586^T	NZ_CP014205
P. grimontii	DSM 17515^T	VFES01000000
P. guariconensis	LMG 27394^T	FMYX01000000
P. huaxiensis	WCHPs060044^T	QKVL01000000
P. humi	CCA1^T	BDGS01000000
P. hutmensis	xwS26^T	QJRG01000000
P. inefficax	JV551A3^T	OPYN01000000
P. japonica	DSM 22348^T	FZOL01000000
P. jinjuensis	NBRC 103047^T	BDAD01000000
P. juntendi	BML3^T	NZ_BLJG01000000
P. kairouanensis	KC12^T	QUZU01000000
P. knackmussii	B13^T	HG322950
P. koreensis	BS3658^T	LT629687
P. kribbensis	46-2^T	CP029608
P. lactis	DSM 29167^T	JYLO01000000
P. libanensis	DSM 17149^T	JYLH01000000
P. linyingensis	LMG 25967^T	FNZE01000000
P. lurida	LMG 21995^T	PDJB01000000

**Species**	**Strains**	**Accession No.**
P. marginalis	DSM 13142^{T}	VFEQ01000000
P. monteilii	NBRC 103158^{T}	JHYV01000000
P. moraviensis	LMG 24280^{T}	NZ_LT629788
P. mosselii	DSM 17497^{T}	JHYW01000000
P. nabeulensis	E10B^{T}	QUZT01000000
P. nitroreducens	NBRC 12694^{T}	BDAI01000000
P. orientalis	DSM 17489^{T}	JYLM01000000
P. otitidis	DSM 17224^{T}	FOJP01000000
P. palleroniana	LMG 23076^{T}	PYWX01000000
P. panacis	DSM 18529^{T}	VFER01000000
P. panipatensis	CCM 7469^{T}	FNDS01000000
P. parafulva	NBRC 16636^{T}	BBIU01000000
P. paralactis	DSM 29164^{T}	JYLN01000000
P. persica	Kh13^{T}	OLKL01000000
P. plecoglossicida	NBRC 103162^{T}	BBIV01000000
P. poae	DSM 14936^{T}	JYLI01000000
P. protegens	CHA0^{T}	CP003190
P. putida	NBRC 14164^{T}	AP013070
P. qingdanensis	JJ3^{T}	PHTD01000000
P. reidholzensis	CCOS 865^{T}	UNOZ01000000
P. resinovorans	DSM 21078^{T}	AUIE01000000
P. rhodesiae	DSM 14020^{T}	VFEU01000000
P. sagittaria	JCM 18195^{T}	FOXM01000000
P. salomonii	ICMP 14252^{T}	FNOX01000000
P. saponiphila	DSM 9751^{T}	FNTJ01000000
P. sichuanensis	WCHPs060039^{T}	QKVM01000000
P. simiae	CCUG 59088^{T}	FOKB01000000
P. soli	LMG 27941^{T}	FOEQ01000000
P. synxantha	NCTC 10696^{T}	LR590482
P. taiwanensis	DSM 21245^{T}	AUEC01000000
P. tohonis	TUM18999^{T}	NZ_AP023189
P. tolaasii	NCPPB 2192^{T}	PHHD01000000
P. trivialis	LMG 21464^{T}	MDFJ01000000
P. tructae	SNU WT1^{T}	CP035952
P. veronii	DSM 11331^{T}	JYLL01000000
P. vranovensis	DSM 16006^{T}	AUED01000000
P. wadenswilerensis	CCOS 864^{T}	UIDD01000000

Table S3. Bacterial identification based on ANI and dDDH analysis using whole genome sequencing

Isolate	Species	ANI[a]	dDDH[a]
BML-PP010	*P. carnis*	95.21	62.8
BML-PP011	*P. rhodesiae*	97.74	80.9
BML-PP012	*P. carnis*	98.18	85.1
BML-PP013	*P. qingdaonensis*	99.23	93.6
BML-PP014[T]	Unidentified	-[b]	-
BML-PP015[T]	Unidentified	-	-
BML-PP016	*P. carnis*	95.38	62.8
BML-PP017	*P. atacamensis*	95.30	63.0
BML-PP018	*P. fluva*	99.49	95.9
BML-PP019	*P. protegens*	98.44	86
BML-PP020[T]	Unidentified	-	-
BML-PP021	*P. juntendi*	98.05	83.1
BML-PP022	*P. asiatica*	99.21	93.8
BML-PP023[T]	Unidentified	-	-
BML-PP024	*P. glycinae*	96.49	71
BML-PP025	*P. protegens*	98.83	90
BML-PP026	*P. juntendi*	98.08	85
BML-PP027	*P. qingdaonensis*	99.23	93.8
BML-PP028[T]	Unidentified	-	-
BML-PP029	*P. otitidis*	98.2	83.6
BML-PP030[T]	Unidentified	-	-
BML-PP031	*P. rhodesiae*	98.78	89.5
BML-PP033	*P. otitidis*	98.26	83.6
BML-PP034	Unidentified	-	-
BML-PP035	*P. carnis*	95.35	62.9
BML-PP036[T]	Unidentified	-	-
BML-PP037	*P. tohonis*	95.98	66.1
BML-PP038	*P. carnis*	95.3	63.1
BML-PP039	*P. glycinae*	98.63	86.9
BML-PP040	*P. lactis*	98.42	85.5
BML-PP041	*P. putida*	98.29	86.2
BML-PP042[T]	Unidentified	-	-
BML-PP043	Unidentified	-	-
BML-PP044	*P. mosselii*	99.2	92.9
BML-PP045	*P. tohonis*	95.98	66.1
BML-PP046	*P. fulva*	99.39	94.9
BML-PP047	*P. juntendi*	98.42	86.2
BML-PP048[T]	Unidentified	-	-
BML-PP049	Unidentified	-	-
BML-PP050	*P. tohonis*	95.98	66.1
BML-PP051	*P. juntendi*	97.94	83.4
BML-PP052	*P. mosselii*	99.17	93.6

[a]Whole genome sequences of the isolates were compared with those of type strains listed in Table S1. The cut-off values were 95% for ANI and/or 70% for dDDH.

[b]Less than the cut-off values.

Table S4. ANI and dDDH comparison of whole genome sequences among 12 isolates[a]

	BML-PP014^{T}		BML-PP015^{T}		BML-PP020^{T}		BML-PP023^{T}		BML-PP028^{T}		BML-PP030^{T}		BML-PP034		BML-PP036^{T}		BML-PP042^{T}		BML-PP043		BML-PP048^{T}		BML-PP049	
	ANI	dDDH	ANI	dDDH	ANI	dDDH	ANI	dDDH	ANI	dDDH	ANI	dDDH	ANI	dDDH	ANI	dDDH	ANI	dDDH	ANI	dDDH	ANI	dDDH	ANI	dDDH
BML-PP014^{T}	-	-	76.4	20.8	78.8	22.4	93.8	54.2	77.8	21.6	87.3	34.3	76.4	20.8	94.1	56.0	78.4	22.1	76.5	20.9	78.9	22.4	79.0	22.5
BML-PP015^{T}	76.4	20.8	-	-	77.5	21.9	76.3	20.9	77.0	21.7	76.2	21.0	**98.1**[b]	**84.4**[b]	76.4	20.9	77.2	21.4	**98.1**	**84.5**	77.9	21.9	77.9	22.1
BML-PP020^{T}	78.8	22.4	77.5	21.9	-	-	78.5	22.3	84.8	29.1	78.4	22.4	77.4	21.9	78.6	22.4	85.3	30.0	77.4	21.9	86.2	31.4	86.6	31.9
BML-PP023^{T}	93.8	54.2	76.3	20.9	78.5	22.3	-	-	77.7	21.7	87.3	34.6	76.4	20.8	94.4	57.7	78.1	22.0	76.4	20.8	78.7	22.5	78.8	22.4
BML-PP028^{T}	77.8	21.6	77.0	21.7	84.8	29.1	77.7	21.7	-	-	78.2	22.7	76.7	21.3	77.9	21.8	86.7	31.8	76.8	21.5	87.4	33.8	87.7	34.4
BML-PP030^{T}	87.3	34.3	76.2	21.0	78.4	22.4	87.3	34.6	78.2	22.7	-	-	76.2	21.0	87.3	34.2	78.3	22.5	76.1	21.0	78.8	22.6	78.6	22.7
BML-PP034	76.4	20.8	**98.1**	**84.4**	77.4	21.9	76.4	20.8	76.7	21.3	76.2	21.0	-	-	76.4	20.7	77.2	21.3	**99.9**	**99.8**	77.6	21.8	77.9	22.0
BML-PP036^{T}	94.1	56.0	76.4	20.9	78.6	22.4	94.4	57.7	77.9	21.8	87.3	34.2	76.4	20.7	-	-	78.4	22.2	76.4	20.8	78.8	22.6	78.9	22.6
BML-PP042^{T}	78.4	22.1	77.2	21.4	85.3	30.0	78.1	22.0	86.7	31.8	78.3	22.5	77.2	21.3	78.4	22.2	-	-	77.1	21.3	87.6	34.3	87.6	34.2
BML-PP043	76.5	20.9	**98.1**	**84.5**	77.4	21.9	76.4	20.8	76.8	21.5	76.1	21.0	**99.9**	**99.8**	76.4	20.8	77.1	21.3	-	-	77.8	21.8	77.9	22.0
BML-PP048^{T}	78.9	22.4	77.9	21.9	86.2	31.4	78.7	22.5	87.4	33.8	78.8	22.6	77.6	21.8	78.8	22.6	87.6	34.3	77.8	21.8	-	-	**98.1**	**83.4**
BML-PP049	79.0	22.5	77.9	22.1	86.6	31.9	78.8	22.4	87.7	34.4	78.6	22.7	77.9	22.0	78.9	22.6	87.6	34.2	77.9	22.0	**98.1**	**83.4**	-	-

[a]The 12 isolates unidentified by ANI and dDDH analysis compared with type strains as shown in Table S2.

[b]Cells in gray: ≧95% of ANI values and ≧70% of dDDH values, indicating that two isolates belonged a species.

BML-PP015, BML-PP034 and BML-PP043 belonged to one species. BML-PP048 and BML-PP049 belonged to a second species. The remaining 7 isolates belonged to an individual species different from others, respectively.

Table S5. Characteristics of the 9 novel type strains

Characteristic	*P. aeruginosa* group	*P. fluorescens* group				*P. putida* group			
	**BML-PP015^{T}**	**BML-PP014^{T}**	**BML-PP023^{T}**	**BML-PP030^{T}**	**BML-PP036^{T}**	**BML-PP020^{T}**	**BML-PP028^{T}**	**BML-PP042^{T}**	**BML-PP048^{T}**
Cell length (μm)	1.0-2.0	1.0-2.0	1.0-2.0	1.0-2.0	1.0-1.5	1.0-1.5	1.0-2.0	1.0-2.0	1.0-2.0
Cell width (μm)	0.5-0.7	0.5-0.7	0.6-0.8	0.6-0.7	0.8-0.9	0.6-0.9	0.7-0.8	0.8-1.0	0.6-0.8
temperature range (℃)	8-36	4-36	4-36	4-36	4-36	8-36	8-36	8-40	8-36
NaCl range (%)	0-6.0%	0-6.0%	0-6.0%	0-5.0%	0-6.0%	0-6.0%	0-6.0%	0-6.0%	0-6.0%
pH range	pH5.5-9.0	pH6.0-9.0	pH6.0-9.0	pH6.0-8.5	pH6.0-9.0	pH6.0-9.0	pH6.0-9.0	pH6.0-9.0	pH6.0-9.0
Fluorescent pigments:									
King's A	-	-	-	-	-	-	-	+	-
King's B	+	+	+	+	+	+	+	+	+
**API20NE results:**									
Potassium nitrate	+	-	-	-	-	-	-	-	+
L-Tryptophan	-	-	-	-	-	-	-	-	-
Glucose (Fermentation)	-	-	-	-	-	-	-	-	-
L-Arginine	+	+	+	+	+	+	+	w	+
Urea	-	-	-	-	-	-	-	-	-
Esculin	-	-	-	-	-	-	-	-	-
Gelatin	-	+	+	+	+	+	-	-	-
p-Nitrophenyl β-D-Galactopyranoside (β-galactosidase)	-	-	-	-	-	-	-	-	-
Glucose (Assimilation)	+	+	+	+	+	w	+	+	+
L-Arabinose	-	+	+	+	+	-	-	+	-
D-Mannose	-	+	+	w	+	-	+	+	w
D-Mannitol	-	+	+	+	+	-	+	-	-
N-Acetyl-D-glucosamine	-	+	+	+	+	-	-	-	-
Maltose	-	-	-	-	-	-	-	-	-
Potassium glucose	+	+	+	+	+	+	+	+	+
n-Capric acid	+	+	+	+	+	w	+	+	+
Adipic acid	+	-	-	-	-	-	-	-	-
DL-Malic acid	+	+	+	+	+	+	+	+	+
Sodium citrate	+	+	+	+	+	+	+	+	+
Phenylmercuric acetate	+	-	-	-	-	+	+	-	+
**API ZYM results:**									
Alkaline phosphatase	w	w	w	w	w	+	+	+	+
Esterase (C4)	+	+	+	+	+	w	w	w	+
Esterase lipase (C8)	+	+	+	+	+	w	w	w	+
Lipase (C14)	-	-	w	w	w	-	-	-	-
Leucine arylamidase	+	+	+	+	+	+	+	+	+

**Characteristic**	***P. aeruginosa* group**	***P. fluorescens* group**				***P. putida* group**			
	**BML-PP015^{T}**	**BML-PP014^{T}**	**BML-PP023^{T}**	**BML-PP030^{T}**	**BML-PP036^{T}**	**BML-PP020^{T}**	**BML-PP028^{T}**	**BML-PP042^{T}**	**BML-PP048^{T}**
Valine arylamidase	w	w	w	w	w	w	w	w	w
Cystine arylamidase	-	-	-	-	-	-	-	-	-
Trypsin	-	-	w	w	w	w	w	w	-
α-Chymotrypsin	-	-	-	-	-	-	-	-	-
Acid phosphatase	+	w	w	w	w	+	+	+	+
Naphthol AS-BI phosphohydrolase	+	+	+	+	+	+	+	+	+
α-Galactosidase	-	-	-	-	-	-	-	-	-
β-Galactosidase	-	-	-	-	-	w	-	-	-
β-Glucuronidase	-	-	-	-	-	-	-	-	-
α-Glucosidase	-	-	-	-	-	-	-	-	-
β-Glucosidase	-	-	-	-	-	-	-	-	-
N-Acetyl-β-glucosaminidase	-	-	-	-	-	-	-	-	-
α-Mannosidase	-	-	-	-	-	-	-	-	-
α-Fucosidase	-	-	-	-	-	-	-	-	-
**Biolog GN3 results:**									
Dextrin	-	-	-	-	-	-	-	-	-
D-Maltose	-	-	-	-	-	-	-	-	-
D-Trehalose	-	-	-	-	-	-	-	-	-
D-Cellobiose	-	-	-	-	-	-	-	-	-
Gentiobiose	-	-	-	-	-	w	w	-	-
Sucrose	-	-	-	-	-	-	-	-	-
D-Turanose	-	-	-	-	-	-	-	-	-
Stachyose	-	-	-	-	-	-	-	-	-
D-Raffinose	-	-	-	-	-	-	-	-	-
α-D-Lactose	-	-	-	-	-	-	-	-	-
D-Melibiose	-	-	-	-	-	-	w	-	-
β-Methyl-D-glucoside	-	-	-	-	-	-	-	-	-
D-Salicin	-	-	-	-	-	-	-	-	-
N-Acetyl-D-glucosamine	-	+	+	+	+	-	-	-	-
N-Acetyl-β-D-mannosamine	-	-	-	-	-	-	-	-	-
N-Acetyl-D-galactosamine	-	-	-	-	-	-	-	-	-
N-Acetyl-neuraminic acid	-	-	-	-	-	-	-	-	-
α-D-Glucose	w	+	+	+	+	+	+	+	w
D-Mannose	-	+	+	+	+	+	+	w	w
D-Fructose	-	+	+	-	+	+	+	w	w
D-Galactose	-	+	+	+	+	w	w	+	w
3-Methyl Glucose	-	-	-	-	-	w	w	-	w
D-Fucose	w	+	w	+	w	w	w	w	w

Characteristic	*P. aeruginosa* group	*P. fluorescens* group				*P. putida* group			
	BML-PP015^T	BML-PP014^T	BML-PP023^T	BML-PP030^T	BML-PP036^T	BML-PP020^T	BML-PP028^T	BML-PP042^T	BML-PP048^T
L-Fucose	w	w	w	w	w	w	w	w	w
L-Rhamnose	-	-	-	-	-	w	w	-	w
Inosine	-	+	+	+	+	-	w	w	w
D-Sorbitol	-	-	-	-	-	-	-	-	-
D-Mannitol	-	+	+	+	+	-	+	-	-
D-Arabitol	-	w	w	w	w	-	w	-	-
myo-Inositol	-	-	+	-	-	-	-	-	-
Glycerol	w	+	+	+	+	+	+	w	+
D-Glucose-6-PO4	-	-	-	-	-	-	-	-	-
D-Fructose-6-PO4	w	w	w	w	w	w	w	w	+
D-Aspartic acid	-	-	-	-	-	+	-	-	-
D-serine	-	-	-	+	w	+	+	+	+
Gelatin	-	-	-	-	-	-	-	-	-
Glycyl-L-Proline	-	-	-	-	-	-	-	-	-
L-Alanine	w	+	+	+	+	+	w	+	+
L-Arginine	+	+	+	+	+	+	w	+	+
L-Aspartic acid	w	+	+	+	+	+	+	+	+
L-Glutamic acid	+	+	+	+	+	+	+	+	+
L-Histidine	-	-	-	w	-	+	w	+	w
L-Pyroglutamic acid	-	+	+	+	+	+	+	+	+
L-Serine	w	+	+	+	+	+	+	+	+
Pectin	-	-	-	-	-	-	-	-	-
D-Galacturonic acid	-	+	-	w	-	w	w	+	w
L-Galactonic acid lactone	w	+	w	w	w	w	w	+	w
D-Gluconic acid	+	+	+	+	+	+	+	+	+
D-Glucuronic acid	-	+	-	w	-	w	w	+	w
Glucuronamide	w	+	w	+	+	+	+	+	+
Mucic acid	-	+	+	+	+	+	-	+	-
Quinic acid	+	+	+	+	+	+	+	+	+
D-Saccharic acid	-	+	+	+	+	+	-	+	-
p-Hydroxy-phenylacetic acid	+	-	-	-	-	w	-	-	-
Methyl pyruvate	w	w	w	w	w	w	+	+	+
D-Lactic acid methyl ester	-	-	-	-	-	-	-	-	-
L-Lactic acid	+	+	+	+	+	+	+	+	+
Citric acid	+	+	+	+	+	+	+	+	+
α-Keto-glutaric acid	+	w	+	+	+	+	+	+	w
D-Malic acid	-	-	-	-	-	+	w	-	+
L-Malic acid	+	+	+	+	+	+	+	+	+

Characteristic	*P. aeruginosa* group	*P. fluorescens* group				*P. putida* group			
	BML-PP015^T	BML-PP014^T	BML-PP023^T	BML-PP030^T	BML-PP036^T	BML-PP020^T	BML-PP028^T	BML-PP042^T	BML-PP048^T
Bromo-succinic acid	-	-	-	w	-	-	+	-	w
Tween 40	-	-	w	w	w	w	-	w	-
γ-Amino-butyric acid	+	+	+	+	+	+	+	+	+
α-Hydroxy-butyric acid	-	-	-	-	-	w	w	w	w
β-Hydroxy-D, L-butyric acid	w	+	+	+	+	+	w	+	w
α-Keto-butyric acid	-	-	-	-	-	w	w	w	w
Acetoacetic acid	-	-	-	-	-	w	w	w	w
Propionic acid	+	+	+	+	+	+	+	+	+
Acetic acid	+	+	+	+	+	+	+	+	+
Formic acid	-	-	-	-	-	w	w	+	w

Table S6. Cellular fatty acid compositions of the 9 novel type strains

Fatty acids	*P. aeruginosa* group	*P. fluorescens* group				*P. putida* group			
	BML-PP015^T	BML-PP014^T	BML-PP023^T	BML-PP030^T	BML-PP036^T	BML-PP020^T	BML-PP028^T	BML-PP042^T	BML-PP048^T
8 : 0 3-OH	ND	ND	ND	ND	ND	ND	ND	ND	TR
10 : 0	TR	TR	TR	TR	TR	ND	TR	TR	TR
10 : 0 2-OH	ND	ND	TR	ND	TR	ND	ND	ND	ND
10 : 0 3-OH	3.7	3.9	3.3	3.1	3.2	9.6	2.4	2.7	3.2
11 : 0	ND	ND	ND	ND	ND	ND	ND	ND	ND
11 : 0 3-OH	TR	ND	ND	ND	ND	TR	ND	ND	ND
11 : 0 iso3-OH	TR	ND	ND	ND	ND	TR	ND	ND	ND
12 : 0	3.2	2.4	2.4	1.6	1.7	1.6	1.2	1.5	1.2
12 : 0 2-OH	7.7	6.3	5.5	6	5.9	5.4	5.2	5.4	5.6
12 : 0 3-OH	5.5	4.7	4	4.1	3.8	4.3	4	4.3	4.3
12 : 1 3-OH	ND	TR	TR	TR	TR	1.0	ND	ND	TR
13 : 0	ND	ND	ND	ND	ND	ND	ND	ND	ND
13 : 0 iso	ND	ND	ND	ND	ND	ND	ND	ND	ND
14 : 0	1.5	TR	TR	TR	TR	TR	1.3	TR	1.5
14 : 1 w5c	TR	ND	ND	ND	ND	ND	ND	ND	ND
15 : 0 iso	TR	ND	ND	ND	ND	ND	ND	ND	ND
15 : 1 w6c	ND	ND	ND	ND	ND	ND	ND	ND	ND
15 : 1 w8c	ND	ND	ND	ND	ND	ND	ND	ND	ND
15 : 1 iso w9c	ND	TR	ND	ND	ND	ND	ND	ND	TR
16 : 0	22.5	28.8	31.4	31.6	31.9	27.7	42.3	40.0	42.2
16 : 0 2-OH	ND	ND	ND	ND	ND	TR	ND	ND	ND
16 : 0 3-OH	ND	TR	ND	ND	ND	ND	ND	ND	ND
16 : 0 iso	TR	ND	ND	ND	ND	ND	ND	ND	ND
16 : 1ω5*c*	TR	TR	TR	TR	TR	ND	TR	TR	TR
16 : 1 2-OH	ND	ND	ND	ND	ND	TR	ND	ND	ND
17 : 0	TR	ND	ND	TR	ND	TR	TR	TR	TR
17 : 0 cyclo	TR	4.7	2.8	3.5	2.8	1.4	11.9	7.4	19.1
17 : 0 iso	TR	ND	ND	ND	ND	ND	TR	TR	ND
17 : 1ω8*c*	TR	ND	ND	ND	ND	TR	ND	ND	ND
18 : 0	TR	TR	TR	TR	TR	TR	1.2	TR	TR
18 : 1ω5*c*	ND	TR	ND	TR	ND	ND	ND	ND	ND
18 : 1ω7*c* 11-methyl	ND	TR	TR	ND	ND	ND	ND	ND	ND
19 : 0 cyclo ω8*c*	TR	TR	TR	TR	ND	ND	TR	TR	TR
19 : 0 iso	ND	ND	ND	ND	ND	ND	ND	ND	TR
19 : 0 10-methyl	TR	ND	ND	ND	ND	ND	ND	ND	ND
Summed feature*									
2	ND	TR	TR	TR	TR	TR	ND	ND	ND
3	27.4	37.5	39.3	37.8	40.3	32.6	17.5	23.4	12.0
8	26.0	9.9	9.9	10.7	8.9	15.0	11.6	13.0	7.9

Values are percentages of total fatty acids. TR, Trace (<1 %); ND, not detected.

* summed feature 2, one or more of C12:0 aldehyde or unknown ECL (equivalent chain lengths) 10.928, isoC16:1 I and C14:0 3-OH; summed feature 3, $C_{16:1}\omega 7c$/$C_{16:1}\omega 6c$; summed feature 8, $C_{18:1}\omega 7c$/$C_{18:1}\omega 6c$.

Table S7. MIC range, MIC50 and MIC90 against the 42 isolates

Agent	MIC (μg/mL)			
	Breakpoint[a]	Range	MIC_{50}	MIC_{90}
Amikacin	64	1-8	2	4
Arbekacin	None	0.25-2	0.5	1
Aztreonam	32	8-256	64	128
Cefepime	None	2-16	4	16
Ceftazidime	32	1-16	4	16
Ciprofloxacin	None	<0.03125-0.5	0.0625	0.125
Colistin	4	0.25-4096	0.5	32
Gentamicin	16	0.25-2	0.5	2
Imipenem	8	0.25-8	1	4
Levofloxacin	8	<0.0625-2	0.5	1
Meropenem	8	0.125-16	2	8
Tigecycline	None	0.5-8	2	4
Tobramycin	16	0.25-2	0.5	1

[a]According to CLSI M100-S25 guideline.

Table S8. Detection of drug resistant genes in the 42 isolates

Isolate	Species	Resistant genes
BML-PP029	*P. otitidis*	*bla*$_{POM-1}$ like
BML-PP030^{T}	*P. parakoreensis* sp. nov.	*aadA6*
BML-PP033	*P. otitidis*	*bla*$_{POM-1}$ like

Description of nine novel species

1) Description of *Pseudomonas sputi* sp. nov.

Pseudomonas sputi (spu'ti. L. gen. n. sputi of sputum).

The cells of this species are aerobic, Gram-negative, non-spore-forming, motile and rod-shaped, measuring 1.0-2.0 μm in length and 0.5-0.7 μm in width. Colonies on Luria broth agar were circular, convex in shape with a creamy color and usually 1-3 mm in diameter after growth for 2 days at 30 °C. These bacteria grew at temperatures between 4-36 °C, at pH 6.0-9.0 and in the presence of 0-6.0 % (w/v) NaCl. They produced catalase and cytochrome oxidase, as well as fluorescent pigments, on King's B agar at 30 °C. On API 20NE tests, BML-PP014^T was positive for L-arginine, gelatin, glucose, L-arabinose, D-mannose, D-mannitol, *N*-acetyl-D-glucosamine, potassium glucose, n-capric acid, DL-malic acid and sodium citrate. On API ZYM tests, this strain was positive for alkaline phosphatase, esterase (C4), esterase lipase (C8), leucine arylamidase, valine arylamidase, acid phosphatase and naphthol-AS-BI-phosphohydrolase activities. On Biolog GN3 tests, this strain was positive for *N*-acetyl-D-glucosamine, α-D-glucose, D-mannose, D-fructose, D-galactose, D-fucose, L-fucose, inosine, D-mannitol, D-arabitol, glycerol, D-fructose-6-PO_4, L-alanine, L-arginine, L-aspartic acid, L-glutamic acid, L-pyroglutamic acid, L-serine, D-galacturonic acid, L-galactonic acid lactone, D-gluconic acid, D-glucuronic acid, glucuronamide, mucic acid, quinic acid, D-saccharic acid, methyl pyruvate, L-lactic acid, citric acid, α-keto-glutaric acid, L-malic acid, γ-amino-butyric acid, β-hydroxy-D, L-butyric acid, propionic acid and acetic acid. The major fatty acids are summed feature 3 ($C_{16:1}$*ω7c*/$C_{16:1}$*ω6c*; 37.5 %), $C_{16:0}$ (28.8 %) and summed feature 8 ($C_{18:1}$*ω7c*/$C_{18:1}$*ω6c*; 9.9 %). The type strain is

BML-PP014^{T} (=JCM 34577^{T}, =LMG 32246^{T}), isolated from a sputum sample of a patient in Japan. The G+C content of the type strain is 60.30 mol%.

2) Description of *Pseudomonas pseudonitroreducens* sp. nov.

Pseudomonas pseudonitroreducens (pseudês, Gr. masc./fem. adj., false; nitro.re.du'cens. L. n. nitrum nitrate; L. part. adj. reducens converting to a different state; N. L. adj. nitroreducens reducing nitrate; pseudonitroreducens, N.L. masc. adj., a false (*Pseudomonas*) *nitroreducens*).

The cells of this species are aerobic, Gram-negative, non-spore-forming, motile and rod-shaped, being 1.0-2.0 µm in length and 0.5-0.7 µm in width. Colonies on Luria broth agar were circular, convex in shape with a creamy color and usually 1-1.5 mm in diameter after growth for 2 days at 30 °C. These bacteria grew at temperatures between 8-36 °C, at pH 5.5-9.0 and in the presence of 0-6.0 % (w/v) NaCl. They produced catalase and cytochrome oxidase, as well as fluorescent pigments, on King's B agar at 30 °C. On API 20NE tests, BML-PP015^{T} was positive for potassium nitrate, L-arginine, glucose, potassium glucose, n-capric acid, adipic acid, DL-malic acid, sodium citrate and phenylmercuric acetate. On API ZYM tests, this strain was positive for alkaline phosphatase, esterase (C4), esterase lipase (C8), leucine arylamidase, valine arylamidase, acid phosphatase and naphthol-AS-BI-phosphohydrolase activities. On Biolog GN3 tests, this strain was positive for α-D-glucose, D-fucose, L-fucose, glycerol, D-fructose-6-PO_4, L-alanine, L-arginine, L-aspartic acid, L-glutamic acid, L-serine, L-galactonic acid lactone, D-gluconic acid, glucuronamide, quinic acid, p-hydroxy-phenylacetic acid, methyl pyruvate, L-lactic acid, citric acid, α-keto-glutaric acid, L-malic acid, γ-amino-butyric acid, β-hydroxy-D, L-butyric acid, propionic acid and acetic

acid. The major fatty acids are summed feature 3 ($C_{16:1}\omega7c$/$C_{16:1}\omega6c$; 27.4 %), summed feature 8 ($C_{18:1}\omega7c$/$C_{18:1}\omega6c$; 26.0 %), and $C_{16:0}$ (22.5 %). The type strain is BML-PP015^T (=JCM 34578^T, =LMG 32247^T), isolated from a sputum sample of a patient in Japan. The G+C content of the type strain is 64.50 mol%.

3) Description of *Pseudomonas parasichuanensis* sp. nov.

Pseudomonas parasichuanensis (pa.ra. Gr. prep. beside, alongside, near, like; si.chuan.en'sis. N.L. fem. adj. referring to Sichuan Province, PR China and the specific epithet of a *Pseudomonas* species; parasichuanensis. N.L. masc. adj. next to (*Pseudomonas*) *sichuanensis*).

The cells of this species are aerobic, Gram-negative, non-spore-forming, motile and rod-shaped, being 1.0-1.5 µm in length and 0.6-0.9 µm in width. Colonies on Luria broth agar were circular, convex in shape with a creamy color and usually 1-2 mm in diameter after growth for 2 days at 30 °C. These bacteria grew at temperatures between 8-36 °C, at pH 6.0-9.0 and in the presence of 0-6.0 % (w/v) NaCl. They produced catalase and cytochrome oxidase, as well as fluorescent pigments, on King's B agar at 30 °C. On API 20NE tests, BML-PP020^T was positive for L-arginine, gelatin, glucose, potassium gluconate, n-capric acid, DL-malic acid, sodium citrate and phenylmercuric acetate. On API ZYM tests, the strain was positive for alkaline phosphatase, esterase (C4), esterase lipase (C8), leucine arylamidase, valine arylamidase, trypsin, acid phosphatase, naphthol-AS-BI-phosphohydrolase and β-galactosidase activities. On Biolog GN3 tests, this strain was positive for gentiobiose, α-D-glucose, D-mannose, D-fructose, D-galactose, 3-methyl glucose, D-fucose, L-fucose, L-rhamnose, glycerol, D-fructose-6-PO_4, D-aspartic acid, D-serine, L-alanine, L-arginine, L-aspartic acid, L-

glutamic acid, L-histidine, L-pyroglutamic acid, L-serine, D-galacturonic acid, L-galactonic acid lactone, D-gluconic acid, D-glucuronic acid, glucuronamide, mucic acid, quinic acid, D-saccharic acid, p-hydroxy-phenylacetic acid, methyl pyruvate, L-lactic acid, citric acid, α-keto-glutaric acid, D-malic acid, L-malic acid, tween 40, γ-amino-butyric acid, α-hydroxy-butyric acid, β-hydroxy-D, L-butyric acid, α-keto-butyric acid, acetoacetic acid, propionic acid, acetic acid and formic acid. The major fatty acids are summed feature 3 ($C_{16:1}\omega 7c$/$C_{16:1}\omega 6c$; 32.6 %), $C_{16:0}$ (27.7 %) and summed feature 8 ($C_{18:1}\omega 7c$/$C_{18:1}\omega 6c$; 15.0 %). The type strain is BML-PP020^{T} (=JCM 34580^{T}, =LMG 32249^{T}), isolated from a vaginal discharge sample of a patient in Japan. The G+C content of the type strain is 64.19 mol%.

4) Description of *Pseudomonas paraglycinae* sp. nov.

Pseudomonas paraglycinae (pa.ra. Gr. prep. beside, alongside, near, like; gly.ci'nae. N.L. gen. n. glycinae of Glycine max, soybean and the specific epithet of a *Pseudomonas* species; paraglycinae. N.L. masc. adj. next to (*Pseudomonas*) *glycinae*). The cells of this species are aerobic, Gram-negative, non-spore-forming, motile and rod-shaped, being 1.0-2.0 μm in length and 0.6-0.8 μm in width. Colonies on Luria broth agar were circular, convex in shape with a creamy color and usually 2-4 mm in diameter after growth for 2 days at 30 °C. These bacteria grew at temperatures between 4-36 °C, at pH 6.0-9.0 and in the presence of 0-6.0 % (w/v) NaCl. They produced catalase and cytochrome oxidase, as well as fluorescent pigments, on King's B agar at 30 °C. On API 20NE tests, BML-PP023^{T} was positive for L-arginine, gelatin, glucose, L-arabinose, D-mannose, D-mannitol, *N*-acetyl-D-glucosamine, potassium gluconate, n-capric acid, DL-malic acid and sodium citrate. On API ZYM tests, the strain was positive

for alkaline phosphatase, esterase (C4), esterase lipase (C8), lipase (C14), leucine arylamidase, valine arylamidase, trypsin, acid phosphatase and naphthol-AS-BI-phosphohydrolase activities. On Biolog GN3 tests, this strain was positive for *N*-acetyl-D-glucosamine, α-D-glucose, D-mannose, D-fructose, D-galactose, D-fucose, L-fucose, inosine, D-mannitol, D-arabitol, myo-inositol, glycerol, D-fructose-6-PO_4, L-alanine, L-arginine, L-aspartic acid, L-glutamic acid, L-pyroglutamic acid, L-serine, L-galactonic acid lactone, D-gluconic acid, glucuronamide, mucic acid, quinic acid, D-saccharic acid, methyl pyruvate, L-lactic acid, citric acid, α-keto-glutaric acid, L-malic acid, tween 40, γ-amino-butyric acid, β-hydroxy-D, L-butyric acid, propionic acid and acetic acid. The major fatty acids are summed feature 3 ($C_{16:1}\omega 7c$/$C_{16:1}\omega 6c$; 39.3 %), $C_{16:0}$ (31.4 %), and summed feature 8 ($C_{18:1}\omega 7c$/$C_{18:1}\omega 6c$; 9.9 %). The type strain is BML-PP023^T (=JCM 34581^T, =LMG 32250^T), isolated from a sputum sample of a patient in Japan. The G+C content of the type strain is 60.68 mol%.

5) Description of *Pseudomonas ceruminis* sp. nov.

Pseudomonas ceruminis (ceru'mi.nis L. gen. neut.n. ceruminis of cerumen).

The cells of this species are aerobic, Gram-negative, non-spore-forming, motile and rod-shaped, being 1.0-2.0 μm in length and 0.7-0.8 μm in width. Colonies on Luria broth agar were circular, convex in shape with a creamy color and usually 1-2 mm in diameter after growth for 2 days at 30 °C. These bacteria grew at temperatures between 8-36 °C, at pH 6.0-9.0 and in the presence of 0-6.0 % (w/v) NaCl. They produced catalase and cytochrome oxidase, as well as fluorescent pigments, on King's B agar at 30 °C. On API 20NE tests, BML-PP028^T was positive for L-arginine, glucose, D-mannose, D-mannitol, potassium glucose, n-capric acid, DL-malic acid, sodium citrate

and phenylmercuric acetate. On API ZYM tests, the strain was positive for alkaline phosphatase, esterase (C4), esterase lipase (C8), leucine arylamidase, valine arylamidase, trypsin, acid phosphatase and naphthol-AS-BI-phosphohydrolase activities. On Biolog GN3 tests, this strain was positive for gentiobiose, D-melibiose, α-D-glucose, D-mannose, D-fructose, D-galactose, 3-methyl glucose, D-fucose, L-fucose, L-rhamnose, inosine, D-mannitol, D-arabitol, glycerol, D-fructose-6-PO_4, D-serine, L-alanine, L-arginine, L-aspartic acid, L-glutamic acid, L-histidine, L-pyroglutamic acid, L-serine, D-galacturonic acid, L-galactonic acid lactone, D-gluconic acid, D-glucuronic acid, glucuronamide, quinic acid, methyl pyruvate, L-lactic acid, citric acid, α-keto-glutaric acid, D-malic acid, L-malic acid, bromo-succinic acid, γ-amino-butyric acid, α-hydroxy-butyric acid, β-hydroxy-D, L-butyric acid, α-keto-butyric acid, acetoacetic acid, propionic acid, acetic acid and formic acid. The major fatty acids are $C_{16:0}$ (42.3 %), summed feature 3 ($C_{16:1}\omega 7c$/$C_{16:1}\omega 6c$; 17.5 %), and $C_{17:0\,cyclo}$ (11.9 %). The type strain is BML-PP028^T (=JCM 34110^T, =DSM 111127^T), isolated from an ear discharge sample of a patient in Japan. The G+C content of the type strain is 61.67 mol%.

6) Description of *Pseudomonas parakoreensis* sp. nov.

Pseudomonas parakoreensis (pa.ra. Gr. prep. beside, alongside, near, like; ko.re.en'sis N.L. fem. adj. koreensis, pertaining to Korea and the specific epithet of a *Pseudomonas* species; parakoreensis. N.L. masc. adj. next to (*Pseudomonas*) *koreensis*).

The cells of this species are aerobic, Gram-negative, non-spore-forming, motile and rod-shaped, being 1.0-2.0 μm in length and 0.6-0.7 μm in width. Colonies on Luria broth agar were circular, convex in shape with a creamy color and usually 1-3 mm in diameter after growth for 2 days at 30 °C. These bacteria grew at temperatures between

4-36 °C, at pH 6.0-8.5 and in the presence of 0-5.0 % (w/v) NaCl. They produced catalase and cytochrome oxidase, as well as fluorescent pigments, on King's B agar at 30 °C. On API 20NE tests, BML-PP030^T was positive for L-arginine, gelatin, glucose, L-arabinose, D-mannose, D-mannitol, *N*-acetyl-D-glucosamine, potassium glucose, n-capric acid, DL-malic acid and sodium citrate. On API ZYM tests, the strain was positive for alkaline phosphatase, esterase (C4), esterase lipase (C8), lipase (C14), leucine arylamidase, valine arylamidase, trypsin, acid phosphatase and naphthol-AS-BI-phosphohydrolase activities. On Biolog GN3 tests, this strain was positive for *N*-acetyl-D-glucosamine, α-D-glucose, D-mannose, D-galactose, D-fucose, L-fucose, inosine, D-mannitol, D-arabitol, glycerol, D-fructose-6-PO_4, D-serine, L-alanine, L-arginine, L-aspartic acid, L-glutamic acid, L-histidine, L-pyroglutamic acid, L-serine, D-galacturonic acid, L-galactonic acid lactone, D-gluconic acid, D-glucuronic acid, glucuronamide, mucic acid, quinic acid, D-saccharic acid, methyl pyruvate, L-lactic acid, citric acid, α-keto-glutaric acid, L-malic acid, bromo-succinic acid, tween 40, γ-amino-butyric acid, β-hydroxy-D, L-butyric acid, propionic acid and acetic acid. The major fatty acids are summed feature 3 ($C_{16:1}\omega 7c$/$C_{16:1}\omega 6c$; 37.8 %), $C_{16:0}$ (31.6 %), and summed feature 8 ($C_{18:1}\omega 7c$/$C_{18:1}\omega 6c$; 10.7 %). The type strain is BML-PP030^T (=JCM 34582^T, =LMG 32251^T), isolated from a throat swab sample of a patient in Japan. The G+C content of the type strain is 60.09 mol%.

7) Description of *Pseudomonas pharyngis* sp. nov.

Pseudomonas pharyngis (pha.ryn'gis Gr. n. phaynx throat; Gr. Gen. n. pharyngis of the throat).

The cells of this species are aerobic, Gram-negative, non-spore-forming, motile and rod-shaped, being 1.0-1.5 µm in length and 0.8-0.9 µm in width. Colonies on Luria broth agar were circular, convex in shape with a creamy color and usually 1-5 mm in diameter after growth for 2 days at 30 °C. These bacteria grew at temperatures between 4-36 °C, at pH 6.0-9.0 and in the presence of 0-6.0 % (w/v) NaCl. They produced catalase and cytochrome oxidase, as well as fluorescent pigments, on King's B agar at 30 °C. On API 20NE tests, BML-PP036^T was positive for L-arginine, gelatin, glucose, L-arabinose, D-mannose, D-mannitol, *N*-acetyl-D-glucosamine, potassium glucose, n-capric acid, DL-malic acid and sodium citrate. On API ZYM tests, the strain was positive for alkaline phosphatase, esterase (C4), esterase lipase (C8), lipase (C14), leucine arylamidase, valine arylamidase, trypsin, acid phosphatase and naphthol-AS-BI-phosphohydrolase activities. On Biolog GN3 tests, this strain was positive for *N*-acetyl-D-glucosamine, α-D-glucose, D-mannose, D-fructose, D-galactose, D-fucose, L-fucose, inosine, D-mannitol, D-arabitol, glycerol, D-fructose-6-PO_4, D-serine, L-alanine, L-arginine, L-aspartic acid, L-glutamic acid, L-pyroglutamic acid, L-serine, L-galactonic acid lactone, D-gluconic acid, glucuronamide, mucic acid, quinic acid, D-saccharic acid, methyl pyruvate, L-lactic acid, citric acid, α-keto-glutaric acid, L-malic acid, tween 40, γ-amino-butyric acid, β-hydroxy-D, L-butyric acid, propionic acid and acetic acid. The major fatty acids are summed feature 3 ($C_{16:1}\omega 7c$/$C_{16:1}\omega 6c$; 40.3 %), $C_{16:0}$ (31.9 %), and summed feature 8 ($C_{18:1}\omega 7c$/$C_{18:1}\omega 6c$; 8.9 %). The type strain is BML-PP036^T (=JCM 34583^T, =LMG 32252^T), isolated from a throat swab sample of a patient in Japan. The G+C content of the type strain is 60.42 mol%.

8) Description of *Pseudomonas urethralis* sp. nov.

Pseudomonas urethralis (L. fem. n. urethra, the excretory canal of the urine, the urethra; L. fem. suff. -alis, suffix denoting pertaining to; N.L. fem. adj. urethralis, of or pertaining to the urethra).

The cells of this species are aerobic, Gram-negative, non-spore-forming, motile and rod-shaped, being 1.0-2.0 μm in length and 0.8-1.0 μm in width. Colonies on Luria broth agar were circular, convex in shape with a creamy color and usually 1-3 mm in diameter after growth for 2 days at 30 °C. These bacteria grew at temperatures between 8-40 °C, at pH 6.0-9.0 and in the presence of 0-6.0 % (w/v) NaCl. They produced catalase and cytochrome oxidase, as well as fluorescent pigments, on King's A and B agar at 30 °C. On API 20NE tests, BML-PP042^T was positive for L-arginine, glucose, L-arabinose, D-mannose, potassium glucose, n-capric acid, DL-malic acid and sodium citrate. On API ZYM tests, the strain was positive for alkaline phosphatase, esterase (C4), esterase lipase (C8), leucine arylamidase, valine arylamidase, trypsin, acid phosphatase and naphthol-AS-BI-phosphohydrolase. On Biolog GN3 tests, this strain was positive for α-D-glucose, D-mannose, D-fructose, D-galactose, D-fucose, L-fucose, inosine, glycerol, D-fructose-6-PO_4, D-serine, L-alanine, L-arginine, L-aspartic acid, L-glutamic acid, L-histidine, L-pyroglutamic acid, L-serine, D-galacturonic acid, L-galactonic acid lactone, D-gluconic acid, D-glucuronic acid, glucuronamide, mucic acid, quinic acid, D-saccharic acid, methyl pyruvate, L-lactic acid, citric acid, α-keto-glutaric acid, L-malic acid, tween 40, γ-amino-butyric acid, α-hydroxy-butyric acid, β-hydroxy-D, L-butyric acid, α-keto-butyric acid, acetoacetic acid, propionic acid, acetic acid and formic acid. The major fatty acids are $C_{16:0}$ (40.0 %), summed feature 3 ($C_{16:1}\omega 7c$/$C_{16:1}\omega 6c$; 23.4 %), and summed feature 8 ($C_{18:1}\omega 7c$/$C_{18:1}\omega 6c$; 13.0 %). The type strain is

BML-PP042^{T} (=JCM 34111^{T}, =DSM 111128^{T}), isolated from a urethral discharge sample of a patient in Japan. The G+C content of the type strain is 62.71 mol%.

9) Description of *Pseudomonas faucium* sp. nov.

Pseudomonas faucium (fau'ci.um L. gen. pl. n. faucium, of the throat).

The cells of this species are aerobic, Gram-negative, non-spore-forming, motile and rod-shaped, being 1.0-2.0 μm in length and 0.6-0.8 μm in width, Colonies on Luria broth agar were circular, convex in shape with a creamy color and usually 1-3 mm in diameter after growth for 2 days at 30 °C. These bacteria grew at temperatures between 8-36 °C, at pH 6.0-9.0 and in the presence of 0-6.0 % (w/v) NaCl. They produced catalase and cytochrome oxidase, as well as fluorescent pigments, on King's B agar at 30 °C. On API 20NE tests, BML-PP048^{T} was positive for potassium nitrate, L-arginine, glucose, D-mannose, potassium glucose, n-capric acid, DL-malic acid, sodium citrate and phenylmercuric acetate. On API ZYM tests, the strain was positive for alkaline phosphatase, esterase (C4), esterase lipase (C8), leucine arylamidase, valine arylamidase, acid phosphatase and naphthol-AS-BI-phosphohydrolase. On Biolog GN3 tests, this strain was positive for α-D-glucose, D-mannose, D-fructose, D-galactose, 3-methyl glucose, D-fucose, L-fucose, L-rhamnose, inosine, glycerol, D-fructose-6-PO_4, D-serine, L-alanine, L-arginine, L-aspartic acid, L-glutamic acid, L-histidine, L-pyroglutamic acid, L-serine, D-galacturonic acid, L-galactonic acid lactone, D-gluconic acid, D-glucuronic acid, glucuronamide, quinic acid, methyl pyruvate, L-lactic acid, citric acid, α-keto-glutaric acid, D-malic acid, L-malic acid, bromo-succinic acid, γ-amino-butyric acid, α-hydroxy-butyric acid, β-hydroxy-D, L-butyric acid, α-keto-butyric acid, acetoacetic acid, propionic acid, acetic acid and formic acid. The major fatty acids are

$C_{16:0}$ (42.2 %), $C_{17:0\ cyclo}$ (19.1 %) and summed feature 3 ($C_{16:1}\omega 7c$/$C_{16:1}\omega 6c$; 12.0 %). The type strain is BML-PP048^T (=JCM 34112^T, =DSM 111130^T), isolated from a throat swab sample of a patient in Japan. The G+C content of the type strain is 64.19 mol%.
