# Supplementary material for: Correction for Tohya et al., “Whole-Genome Sequencing-Based Re-Identification of Pseudomonas putida/fluorescens Clinical Isolates Identified by Biochemical Bacterial Identification Systems”
Source: Microbiol Spectr. 2023 Feb 21;11(2):e04305-22. doi: 10.1128/spectrum.04305-22 (PMC10100977; doi:10.1128/spectrum.04305-22)
Supplement: Supplemental file 1 — Fig. S1, Table S1 to S8. Download spectrum.04305-22-s0001.pdf, PDF file, 0.5 MB [file spectrum.04305-22-s0001.pdf]

Microbiology Spectrum
Supplemental Materials

Correction for Tohya et al., "Whole-Genome Sequencing-Based Re-Identification of *Pseudomonas putida/fluorescens* Clinical Isolates Identified by Biochemical Bacterial Identification Systems"

Mari Tohya[1, 2], Kanae Teramoto[3], Shin Watanabe[2], Tomomi Hishinuma[1], Masahito Shimojima[4,5], Miho Ogawa[5], Tatsuya Tada[1], Yoko Tabe[6], Teruo Kirikae[1*]

[1]Department of Microbiology, Juntendo University School of Medicine, Tokyo, Japan, [2]Department of Microbiome Research, Juntendo University School of Medicine, Tokyo, Japan, [3]Koichi Tanaka Mass Spectrometry Research Laboratory, Kyoto, Japan, [4]SUGIYAMA-GEN Co., Ltd., Tokyo, Japan, [5]BML, Inc., Saitama, Japan, [6]Department of Clinical Laboratory Medicine, Juntendo University Graduate School of Medicine, Tokyo, Japan

[*]**Corresponding author:** Teruo Kirikae, M.D., Ph.D., t-kirikae@juntendo.ac.jp
Address: Department of Microbiology, Juntendo University School of Medicine, Tokyo, Japan, 2-1-1 Hongo, Bunkyo-ku, Tokyo 113-8421, Japan
Phone: (81) 3 5802 1041, **Fax:** (81) 3 5684 7830

Running Title: Re-identification of *Pseudomonas* isolates

KEYWORDS: *Pseudomonas*, human pathogen, re-identification

Figure S1. Comparative MALDI-TOF MS profiles of type strains of novel species and related *Pseudomonas* species.

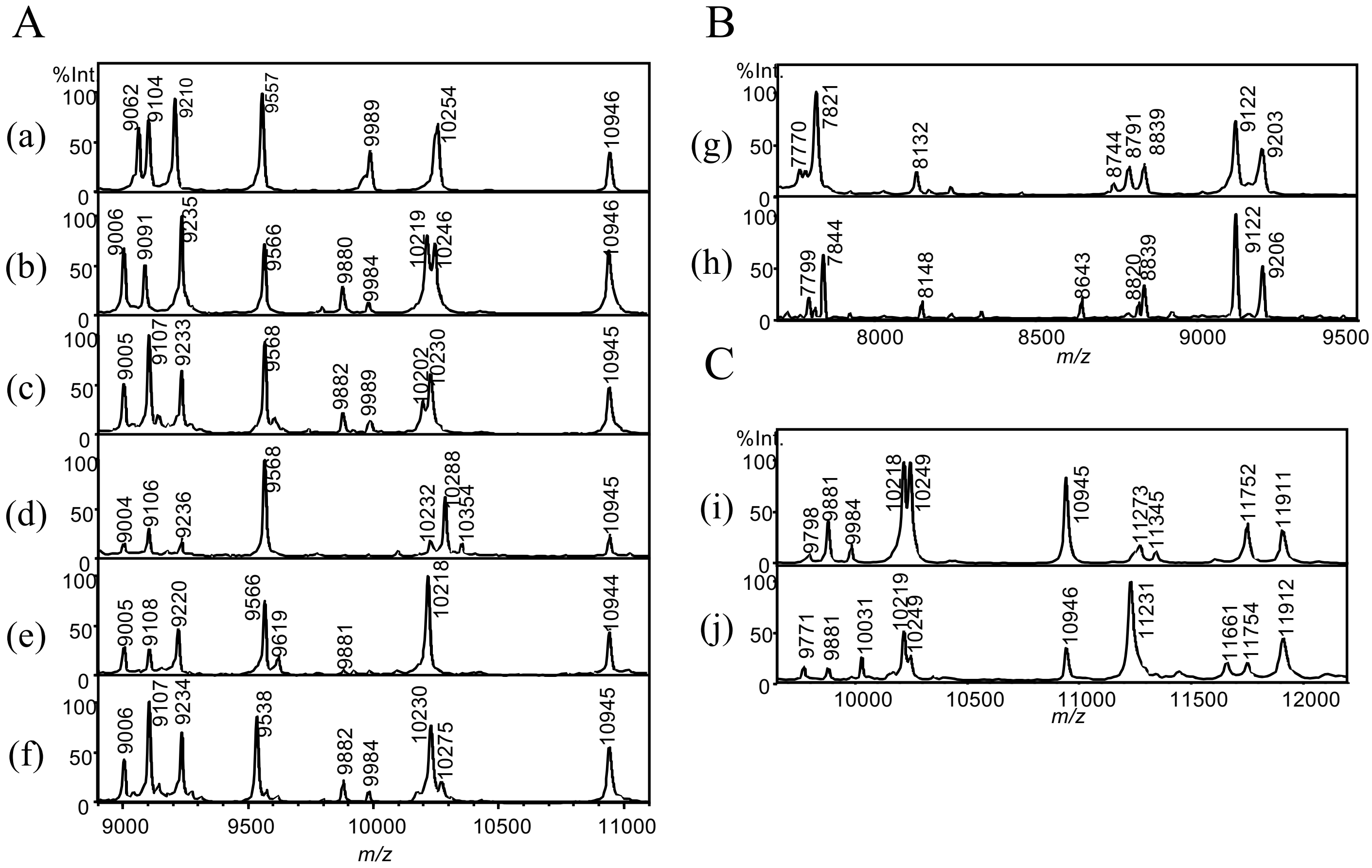

A: MALDI-TOF MS profiles (m/z 9000-11100) of (a) *P. fluorescens* NBRC 14160^{T}, (b) *P. koreensis* JCM 14769^{T}, (c) *P. glycinae* LMG 30275^{T}, (d) *P. sputi* sp. nov. BML-PP014^{T}, (e) *P. paraglycinae* sp. nov. BML-PP023^{T} and (f) *P. pharyngis* sp. nov. BML-PP036^{T}. B: MALDI-TOF MS profiles (m/z 7700-9500) of (g) *P. sichuanensis* JCM 32906^{T} and (h) *P. parasichuanensis* sp. nov. BML-PP020^{T}. C: MALDI-TOF MS profiles (m/z 9700-12200) of (i) *P. koreensis* JCM 32906^{T} and (j) *P. parakoreensis* sp. nov. BML-PP030^{T}.

MALDI-TOF MS detected one to four unique major peaks in each of the five novel species, including at *m/z* 9220, 9619 and 10218 for *P. paraglycinae* sp. nov.; at *m/z* 9771, 10031, 11231 and 11661 for *P. parakoreensis* sp. nov.; at *m/z* 7799, 7844, 8148 and 8643 for *P. parasichuanensis* sp. nov.; at *m/z* 9538 and 10275 for *P. pharyngis* sp. nov.; and at *m/z* 10354 for *P. sputi* sp. nov.

Table S1. Accession numbers of whole genome sequence data for the clinical 42 isolates

Isolate	Accession No.
BML-PP010	BQHE00000000
BML-PP011	BQHF00000000
BML-PP012	BQHG00000000
BML-PP013	BQHH00000000
BML-PP014^{T}	BQHI00000000
BML-PP015^{T}	BQHJ00000000
BML-PP016	BQHK00000000
BML-PP017	BQHL00000000
BML-PP018	BQHM00000000
BML-PP019	BQHN00000000
BML-PP020^{T}	BQHO00000000
BML-PP021	BQHP00000000
BML-PP022	BQHQ00000000
BML-PP023^{T}	BQHR00000000
BML-PP024	BQHS00000000
BML-PP025	BQHT00000000
BML-PP026	BQHU00000000
BML-PP027	BQHV00000000
BML-PP028^{T}	BQHW00000000
BML-PP029	BQHX00000000
BML-PP030^{T}	BQHY00000000
BML-PP031	BQHZ00000000
BML-PP033	BQIA00000000
BML-PP034	BQIB00000000
BML-PP035	BQIC00000000
BML-PP036^{T}	BQID00000000
BML-PP037	BQIE00000000
BML-PP038	BQIF00000000
BML-PP039	BQIG00000000
BML-PP040	BQIH00000000
BML-PP041	BQII00000000
BML-PP042^{T}	BQIJ00000000
BML-PP043	BQIK00000000
BML-PP044	BQIL00000000
BML-PP045	BQIM00000000
BML-PP046	BQIN00000000
BML-PP047	BQIO00000000
BML-PP048^{T}	BQIP00000000
BML-PP049	BQIQ00000000
BML-PP050	BQIR00000000
BML-PP051	BQIS00000000
BML-PP052	BQIT00000000

Table S2. Accession numbers of whole genome sequence data for type strains

Species	Strains	Accession No.
P. aeruginosa	DSM 50071^T	NZ_CP012001
P. agarici	NCPPB 2289^T	AKBQ01000000
P. alcaligenes	NBRC 14159^T	BATI01000000
P. alkylphenolica	KL28^T	CP009048
P. alloputida	Kh7^T	OLKK01000000
P. antarctica	DSM 15318^T	UYXQ01000000
P. asiatica	RYU5^T	NZ_BLJF01000000
P. asplenii	ATCC 23835^T	NZ_LT629777
P. atacamensis	M7D1^T	SSBS01000000
P. azotoformans	DSM 18862^T	MNPV01000000
P. baetica	a390^T	PKLC01000000
P. batumici	UCM B-321^T	JXDG01000000
P. canadensis	2-92^T	AYTD01000000
P. carnis	B4-1^T	NZ_CABIVL01000000
P. cedrina subsp. *cedrina*	DSM 117516^T	UYXV01000000
P. chlororaphis subsp. *chlororaphis*	DSM 50083^T	CP027712
P. citronellolis	NBRC 103043^T	BCZY01000000
P. constantinii	LMG 22119^T	MDDR01000000
P. cremoricolorata	NBRC 16634^T	AUEA01000000
P. delhiensis	CCM 7361^T	FNEC01000000
P. donghuensis	HYS^T	AJJP01000000
P. entomophila	L48^T	CT573326
P. extremaustralis	DSM 17835^T	LT629689
P. extremorientalis	LMG 19695^T	MDGK01000000
P. fluorescens	NCTC 10038^T	LS483372
P. fulva	NBRC 16637^T	BBIQ01000000
P. glycinae	MS586^T	NZ_CP014205
P. grimontii	DSM 17515^T	VFES01000000
P. guariconensis	LMG 27394^T	FMYX01000000
P. huaxiensis	WCHPs060044^T	QKVL01000000
P. humi	CCA1^T	BDGS01000000
P. hutmensis	xwS26^T	QJRG01000000
P. inefficax	JV551A3^T	OPYN01000000
P. japonica	DSM 22348^T	FZOL01000000
P. jinjuensis	NBRC 103047^T	BDAD01000000
P. juntendi	BML3^T	NZ_BLJG01000000
P. kairouanensis	KC12^T	QUZU01000000
P. knackmussii	B13^T	HG322950
P. koreensis	BS3658^T	LT629687
P. kribbensis	46-2^T	CP029608
P. lactis	DSM 29167^T	JYLO01000000
P. libanensis	DSM 17149^T	JYLH01000000
P. linyingensis	LMG 25967^T	FNZE01000000
P. lurida	LMG 21995^T	PDJB01000000

Species	Strains	Accession No.
P. marginalis	DSM 13142^T	VFEQ01000000
P. monteilii	NBRC 103158^T	JHYV01000000
P. moraviensis	LMG 24280^T	NZ_LT629788
P. mosselii	DSM 17497^T	JHYW01000000
P. nabeulensis	E10B^T	QUZT01000000
P. nitroreducens	NBRC 12694^T	BDAI01000000
P. orientalis	DSM 17489^T	JYLM01000000
P. otitidis	DSM 17224^T	FOJP01000000
P. palleroniana	LMG 23076^T	PYWX01000000
P. panacis	DSM 18529^T	VFER01000000
P. panipatensis	CCM 7469^T	FNDS01000000
P. parafulva	NBRC 16636^T	BBIU01000000
P. paralactis	DSM 29164^T	JYLN01000000
P. persica	Kh13^T	OLKL01000000
P. plecoglossicida	NBRC 103162^T	BBIV01000000
P. poae	DSM 14936^T	JYLI01000000
P. protegens	CHA0^T	CP003190
P. putida	NBRC 14164^T	AP013070
P. qingdanensis	JJ3^T	PHTD01000000
P. reidholzensis	CCOS 865^T	UNOZ01000000
P. resinovorans	DSM 21078^T	AUIE01000000
P. rhodesiae	DSM 14020^T	VFEU01000000
P. sagittaria	JCM 18195^T	FOXM01000000
P. salomonii	ICMP 14252^T	FNOX01000000
P. saponiphila	DSM 9751^T	FNTJ01000000
P. sichuanensis	WCHPs060039^T	QKVM01000000
P. simiae	CCUG 59088^T	FOKB01000000
P. soli	LMG 27941^T	FOEQ01000000
P. synxantha	NCTC 10696^T	LR590482
P. taiwanensis	DSM 21245^T	AUEC01000000
P. tohonis	TUM18999^T	NZ_AP023189
P. tolaasii	NCPPB 2192^T	PHHD01000000
P. trivialis	LMG 21464^T	MDFJ01000000
P. tructae	SNU WT1^T	CP035952
P. veronii	DSM 11331^T	JYLL01000000
P. vranovensis	DSM 16006^T	AUED01000000
P. wadenswilerensis	CCOS 864^T	UIDD01000000

Table S3. Bacterial identification based on ANI and dDDH analysis using whole genome sequencing

Isolate	Species	ANI[a]	dDDH[a]
BML-PP010	*P. carnis*	95.21	62.8
BML-PP011	*P. rhodesiae*	97.74	80.9
BML-PP012	*P. carnis*	98.18	85.1
BML-PP013	*P. qingdaonensis*	99.23	93.6
BML-PP014[T]	Unidentified	-[b]	-
BML-PP015[T]	Unidentified	-	-
BML-PP016	*P. carnis*	95.38	62.8
BML-PP017	*P. atacamensis*	95.30	63.0
BML-PP018	*P. fluva*	99.49	95.9
BML-PP019	*P. protegens*	98.44	86
BML-PP020[T]	Unidentified	-	-
BML-PP021	*P. juntendi*	98.05	83.1
BML-PP022	*P. asiatica*	99.21	93.8
BML-PP023[T]	Unidentified	-	-
BML-PP024	*P. glycinae*	96.49	71
BML-PP025	*P. protegens*	98.83	90
BML-PP026	*P. juntendi*	98.08	85
BML-PP027	*P. qingdaonensis*	99.23	93.8
BML-PP028[T]	Unidentified	-	-
BML-PP029	*P. otitidis*	98.2	83.6
BML-PP030[T]	Unidentified	-	-
BML-PP031	*P. rhodesiae*	98.78	89.5
BML-PP033	*P. otitidis*	98.26	83.6
BML-PP034	Unidentified	-	-
BML-PP035	*P. carnis*	95.35	62.9
BML-PP036[T]	Unidentified	-	-
BML-PP037	*P. tohonis*	95.98	66.1
BML-PP038	*P. carnis*	95.3	63.1
BML-PP039	*P. glycinae*	98.63	86.9
BML-PP040	*P. lactis*	98.42	85.5
BML-PP041	*P. putida*	98.29	86.2
BML-PP042[T]	Unidentified	-	-
BML-PP043	Unidentified	-	-
BML-PP044	*P. mosselii*	99.2	92.9
BML-PP045	*P. tohonis*	95.98	66.1
BML-PP046	*P. fulva*	99.39	94.9
BML-PP047	*P. juntendi*	98.42	86.2
BML-PP048[T]	Unidentified	-	-
BML-PP049	Unidentified	-	-
BML-PP050	*P. tohonis*	95.98	66.1
BML-PP051	*P. juntendi*	97.94	83.4
BML-PP052	*P. mosselii*	99.17	93.6

[a]Whole genome sequences of the isolates were compared with those of type strains listed in Table S1. The cut-off values were 95% for ANI and/or 70% for dDDH.

[b]Less than the cut-off values.

Table S4. ANI and dDDH comparison of whole genome sequences among 12 isolates[a]

	BML-PP014^{T}		BML-PP015^{T}		BML-PP020^{T}		BML-PP023^{T}		BML-PP028^{T}		BML-PP030^{T}		BML-PP034		BML-PP036^{T}		BML-PP042^{T}		BML-PP043		BML-PP048^{T}		BML-PP049	
	ANI	dDDH	ANI	dDDH	ANI	dDDH	ANI	dDDH	ANI	dDDH	ANI	dDDH	ANI	dDDH	ANI	dDDH	ANI	dDDH	ANI	dDDH	ANI	dDDH	ANI	dDDH
BML-PP014^{T}	-	-	76.4	20.8	78.8	22.4	93.8	54.2	77.8	21.6	87.3	34.3	76.4	20.8	94.1	56.0	78.4	22.1	76.5	20.9	78.9	22.4	79.0	22.5
BML-PP015^{T}	76.4	20.8	-	-	77.5	21.9	76.3	20.9	77.0	21.7	76.2	21.0	**98.1**[b]	**84.4**[b]	76.4	20.9	77.2	21.4	**98.1**	**84.5**	77.9	21.9	77.9	22.1
BML-PP020^{T}	78.8	22.4	77.5	21.9	-	-	78.5	22.3	84.8	29.1	78.4	22.4	77.4	21.9	78.6	22.4	85.3	30.0	77.4	21.9	86.2	31.4	86.6	31.9
BML-PP023^{T}	93.8	54.2	76.3	20.9	78.5	22.3	-	-	77.7	21.7	87.3	34.6	76.4	20.8	94.4	57.7	78.1	22.0	76.4	20.8	78.7	22.5	78.8	22.4
BML-PP028^{T}	77.8	21.6	77.0	21.7	84.8	29.1	77.7	21.7	-	-	78.2	22.7	76.7	21.3	77.9	21.8	86.7	31.8	76.8	21.5	87.4	33.8	87.7	34.4
BML-PP030^{T}	87.3	34.3	76.2	21.0	78.4	22.4	87.3	34.6	78.2	22.7	-	-	76.2	21.0	87.3	34.2	78.3	22.5	76.1	21.0	78.8	22.6	78.6	22.7
BML-PP034	76.4	20.8	**98.1**	**84.4**	77.4	21.9	76.4	20.8	76.7	21.3	76.2	21.0	-	-	76.4	20.7	77.2	21.3	**99.9**	**99.8**	77.6	21.8	77.9	22.0
BML-PP036^{T}	94.1	56.0	76.4	20.9	78.6	22.4	94.4	57.7	77.9	21.8	87.3	34.2	76.4	20.7	-	-	78.4	22.2	76.4	20.8	78.8	22.6	78.9	22.6
BML-PP042^{T}	78.4	22.1	77.2	21.4	85.3	30.0	78.1	22.0	86.7	31.8	78.3	22.5	77.2	21.3	78.4	22.2	-	-	77.1	21.3	87.6	34.3	87.6	34.2
BML-PP043	76.5	20.9	**98.1**	**84.5**	77.4	21.9	76.4	20.8	76.8	21.5	76.1	21.0	**99.9**	**99.8**	76.4	20.8	77.1	21.3	-	-	77.8	21.8	77.9	22.0
BML-PP048^{T}	78.9	22.4	77.9	21.9	86.2	31.4	78.7	22.5	87.4	33.8	78.8	22.6	77.6	21.8	78.8	22.6	87.6	34.3	77.8	21.8	-	-	**98.1**	**83.4**
BML-PP049	79.0	22.5	77.9	22.1	86.6	31.9	78.8	22.4	87.7	34.4	78.6	22.7	77.9	22.0	78.9	22.6	87.6	34.2	77.9	22.0	**98.1**	**83.4**	-	-

[a]The 12 isolates unidentified by ANI and dDDH analysis compared with type strains as shown in Table S2.

[b]Cells in gray: ≧95% of ANI values and ≧70% of dDDH values, indicating that two isolates belonged a species.

BML-PP015, BML-PP034 and BML-PP043 belonged to one species. BML-PP048 and BML-PP049 belonged to a second species. The remaining 7 isolates belonged to an individual species different from others, respectively.

Table S5. Characteristics of the 9 novel type strains

Characteristic	*P. aeruginosa* group	*P. fluorescens* group				*P. putida* group			
	BML-PP015^T	BML-PP014^T	BML-PP023^T	BML-PP030^T	BML-PP036^T	BML-PP020^T	BML-PP028^T	BML-PP042^T	BML-PP048^T
Cell length (μm)	1.0-2.0	1.0-2.0	1.0-2.0	1.0-2.0	1.0-1.5	1.0-1.5	1.0-2.0	1.0-2.0	1.0-2.0
Cell width (μm)	0.5-0.7	0.5-0.7	0.6-0.8	0.6-0.7	0.8-0.9	0.6-0.9	0.7-0.8	0.8-1.0	0.6-0.8
temperature range (℃)	8-36	4-36	4-36	4-36	4-36	8-36	8-36	8-40	8-36
NaCl range (%)	0-6.0%	0-6.0%	0-6.0%	0-5.0%	0-6.0%	0-6.0%	0-6.0%	0-6.0%	0-6.0%
pH range	pH5.5-9.0	pH6.0-9.0	pH6.0-9.0	pH6.0-8.5	pH6.0-9.0	pH6.0-9.0	pH6.0-9.0	pH6.0-9.0	pH6.0-9.0
Fluorescent pigments:									
King's A	-	-	-	-	-	-	-	+	-
King's B	+	+	+	+	+	+	+	+	+
**API20NE results:**									
Potassium nitrate	+	-	-	-	-	-	-	-	+
L-Tryptophan	-	-	-	-	-	-	-	-	-
Glucose (Fermentation)	-	-	-	-	-	-	-	-	-
L-Arginine	+	+	+	+	+	+	+	w	+
Urea	-	-	-	-	-	-	-	-	-
Esculin	-	-	-	-	-	-	-	-	-
Gelatin	-	+	+	+	+	+	-	-	-
p-Nitrophenyl β-D-Galactopyranoside (β-galactosidase)	-	-	-	-	-	-	-	-	-
Glucose (Assimilation)	+	+	+	+	+	w	+	+	+
L-Arabinose	-	+	+	+	+	-	-	+	-
D-Mannose	-	+	+	w	+	-	+	+	w
D-Mannitol	-	+	+	+	+	-	+	-	-
N-Acetyl-D-glucosamine	-	+	+	+	+	-	-	-	-
Maltose	-	-	-	-	-	-	-	-	-
Potassium glucose	+	+	+	+	+	+	+	+	+
n-Capric acid	+	+	+	+	+	w	+	+	+
Adipic acid	+	-	-	-	-	-	-	-	-
DL-Malic acid	+	+	+	+	+	+	+	+	+
Sodium citrate	+	+	+	+	+	+	+	+	+
Phenylmercuric acetate	+	-	-	-	-	+	+	-	+
**API ZYM results:**									
Alkaline phosphatase	w	w	w	w	w	+	+	+	+
Esterase (C4)	+	+	+	+	+	w	w	w	+
Esterase lipase (C8)	+	+	+	+	+	w	w	w	+
Lipase (C14)	-	-	w	w	w	-	-	-	-
Leucine arylamidase	+	+	+	+	+	+	+	+	+

**Characteristic**	***P. aeruginosa* group**	***P. fluorescens* group**				***P. putida* group**			
	**BML-PP015T**	**BML-PP014T**	**BML-PP023T**	**BML-PP030T**	**BML-PP036T**	**BML-PP020T**	**BML-PP028T**	**BML-PP042T**	**BML-PP048T**
Valine arylamidase	w	w	w	w	w	w	w	w	w
Cystine arylamidase	-	-	-	-	-	-	-	-	-
Trypsin	-	-	w	w	w	w	w	w	-
α-Chymotrypsin	-	-	-	-	-	-	-	-	-
Acid phosphatase	+	w	w	w	w	+	+	+	+
Naphthol AS-BI phosphohydrolase	+	+	+	+	+	+	+	+	+
α-Galactosidase	-	-	-	-	-	-	-	-	-
β-Galactosidase	-	-	-	-	-	w	-	-	-
β-Glucuronidase	-	-	-	-	-	-	-	-	-
α-Glucosidase	-	-	-	-	-	-	-	-	-
β-Glucosidase	-	-	-	-	-	-	-	-	-
N-Acetyl-β-glucosaminidase	-	-	-	-	-	-	-	-	-
α-Mannosidase	-	-	-	-	-	-	-	-	-
α-Fucosidase	-	-	-	-	-	-	-	-	-
**Biolog GN3 results:**									
Dextrin	-	-	-	-	-	-	-	-	-
D-Maltose	-	-	-	-	-	-	-	-	-
D-Trehalose	-	-	-	-	-	-	-	-	-
D-Cellobiose	-	-	-	-	-	-	-	-	-
Gentiobiose	-	-	-	-	-	w	w	-	-
Sucrose	-	-	-	-	-	-	-	-	-
D-Turanose	-	-	-	-	-	-	-	-	-
Stachyose	-	-	-	-	-	-	-	-	-
D-Raffinose	-	-	-	-	-	-	-	-	-
α-D-Lactose	-	-	-	-	-	-	-	-	-
D-Melibiose	-	-	-	-	-	-	w	-	-
β-Methyl-D-glucoside	-	-	-	-	-	-	-	-	-
D-Salicin	-	-	-	-	-	-	-	-	-
N-Acetyl-D-glucosamine	-	+	+	+	+	-	-	-	-
N-Acetyl-β-D-mannosamine	-	-	-	-	-	-	-	-	-
N-Acetyl-D-galactosamine	-	-	-	-	-	-	-	-	-
N-Acetyl-neuraminic acid	-	-	-	-	-	-	-	-	-
α-D-Glucose	w	+	+	+	+	+	+	+	w
D-Mannose	-	+	+	+	+	+	+	w	w
D-Fructose	-	+	+	-	+	+	+	w	w
D-Galactose	-	+	+	+	+	w	w	+	w
3-Methyl Glucose	-	-	-	-	-	w	w	-	w
D-Fucose	w	+	w	+	w	w	w	w	w

Characteristic	*P. aeruginosa* group	*P. fluorescens* group				*P. putida* group			
	BML-PP015^{T}	BML-PP014^{T}	BML-PP023^{T}	BML-PP030^{T}	BML-PP036^{T}	BML-PP020^{T}	BML-PP028^{T}	BML-PP042^{T}	BML-PP048^{T}
L-Fucose	w	w	w	w	w	w	w	w	w
L-Rhamnose	-	-	-	-	-	w	w	-	w
Inosine	-	+	+	+	+	-	w	w	w
D-Sorbitol	-	-	-	-	-	-	-	-	-
D-Mannitol	-	+	+	+	+	-	+	-	-
D-Arabitol	-	w	w	w	w	-	w	-	-
myo-Inositol	-	-	+	-	-	-	-	-	-
Glycerol	w	+	+	+	+	+	+	w	+
D-Glucose-6-PO4	-	-	-	-	-	-	-	-	-
D-Fructose-6-PO4	w	w	w	w	w	w	w	w	+
D-Aspartic acid	-	-	-	-	-	+	-	-	-
D-serine	-	-	-	+	w	+	+	+	+
Gelatin	-	-	-	-	-	-	-	-	-
Glycyl-L-Proline	-	-	-	-	-	-	-	-	-
L-Alanine	w	+	+	+	+	+	w	+	+
L-Arginine	+	+	+	+	+	+	w	+	+
L-Aspartic acid	w	+	+	+	+	+	+	+	+
L-Glutamic acid	+	+	+	+	+	+	+	+	+
L-Histidine	-	-	-	w	-	+	w	+	w
L-Pyroglutamic acid	-	+	+	+	+	+	+	+	+
L-Serine	w	+	+	+	+	+	+	+	+
Pectin	-	-	-	-	-	-	-	-	-
D-Galacturonic acid	-	+	-	w	-	w	w	+	w
L-Galactonic acid lactone	w	+	w	w	w	w	w	+	w
D-Gluconic acid	+	+	+	+	+	+	+	+	+
D-Glucuronic acid	-	+	-	w	-	w	w	+	w
Glucuronamide	w	+	w	+	+	+	+	+	+
Mucic acid	-	+	+	+	+	+	-	+	-
Quinic acid	+	+	+	+	+	+	+	+	+
D-Saccharic acid	-	+	+	+	+	+	-	+	-
p-Hydroxy-phenylacetic acid	+	-	-	-	-	w	-	-	-
Methyl pyruvate	w	w	w	w	w	w	+	+	+
D-Lactic acid methyl ester	-	-	-	-	-	-	-	-	-
L-Lactic acid	+	+	+	+	+	+	+	+	+
Citric acid	+	+	+	+	+	+	+	+	+
α-Keto-glutaric acid	+	w	+	+	+	+	+	+	w
D-Malic acid	-	-	-	-	-	+	w	-	+
L-Malic acid	+	+	+	+	+	+	+	+	+

Characteristic	*P. aeruginosa* group	*P. fluorescens* group				*P. putida* group			
	BML-PP015[T]	BML-PP014[T]	BML-PP023[T]	BML-PP030[T]	BML-PP036[T]	BML-PP020[T]	BML-PP028[T]	BML-PP042[T]	BML-PP048[T]
Bromo-succinic acid	-	-	-	w	-	-	+	-	w
Tween 40	-	-	w	w	w	w	-	w	-
γ-Amino-butyric acid	+	+	+	+	+	+	+	+	+
α-Hydroxy-butyric acid	-	-	-	-	-	w	w	w	w
β-Hydroxy-D, L-butyric acid	w	+	+	+	+	+	w	+	w
α-Keto-butyric acid	-	-	-	-	-	w	w	w	w
Acetoacetic acid	-	-	-	-	-	w	w	w	w
Propionic acid	+	+	+	+	+	+	+	+	+
Acetic acid	+	+	+	+	+	+	+	+	+
Formic acid	-	-	-	-	-	w	w	+	w

Table S6. Cellular fatty acid compositions of the 9 novel type strains

Fatty acids	*P. aeruginosa* group	*P. fluorescens* group				*P. putida* group			
	BML-PP015T	BML-PP014T	BML-PP023T	BML-PP030T	BML-PP036T	BML-PP020T	BML-PP028T	BML-PP042T	BML-PP048T
8 : 0 3-OH	ND	ND	ND	ND	ND	ND	ND	ND	TR
10 : 0	TR	TR	TR	TR	TR	ND	TR	TR	TR
10 : 0 2-OH	ND	ND	TR	ND	TR	ND	ND	ND	ND
10 : 0 3-OH	3.7	3.9	3.3	3.1	3.2	9.6	2.4	2.7	3.2
11 : 0	ND	ND	ND	ND	ND	ND	ND	ND	ND
11 : 0 3-OH	TR	ND	ND	ND	ND	TR	ND	ND	ND
11 : 0 iso3-OH	TR	ND	ND	ND	ND	TR	ND	ND	ND
12 : 0	3.2	2.4	2.4	1.6	1.7	1.6	1.2	1.5	1.2
12 : 0 2-OH	7.7	6.3	5.5	6	5.9	5.4	5.2	5.4	5.6
12 : 0 3-OH	5.5	4.7	4	4.1	3.8	4.3	4	4.3	4.3
12 : 1 3-OH	ND	TR	TR	TR	TR	1.0	ND	ND	TR
13 : 0	ND	ND	ND	ND	ND	ND	ND	ND	ND
13 : 0 iso	ND	ND	ND	ND	ND	ND	ND	ND	ND
14 : 0	1.5	TR	TR	TR	TR	TR	1.3	TR	1.5
14 : 1 w5c	TR	ND	ND	ND	ND	ND	ND	ND	ND
15 : 0 iso	TR	ND	ND	ND	ND	ND	ND	ND	ND
15 : 1 w6c	ND	ND	ND	ND	ND	ND	ND	ND	ND
15 : 1 w8c	ND	ND	ND	ND	ND	ND	ND	ND	ND
15 : 1 iso w9c	ND	TR	ND	ND	ND	ND	ND	ND	TR
16 : 0	22.5	28.8	31.4	31.6	31.9	27.7	42.3	40.0	42.2
16 : 0 2-OH	ND	ND	ND	ND	ND	TR	ND	ND	ND
16 : 0 3-OH	ND	TR	ND	ND	ND	ND	ND	ND	ND
16 : 0 iso	TR	ND	ND	ND	ND	ND	ND	ND	ND
16 : 1ω5*c*	TR	TR	TR	TR	TR	ND	TR	TR	TR
16 : 1 2-OH	ND	ND	ND	ND	ND	TR	ND	ND	ND
17 : 0	TR	ND	ND	TR	ND	TR	TR	TR	TR
17 : 0 cyclo	TR	4.7	2.8	3.5	2.8	1.4	11.9	7.4	19.1
17 : 0 iso	TR	ND	ND	ND	ND	ND	TR	TR	ND
17 : 1ω8*c*	TR	ND	ND	ND	ND	TR	ND	ND	ND
18 : 0	TR	TR	TR	TR	TR	TR	1.2	TR	TR
18 : 1ω5*c*	ND	TR	ND	TR	ND	ND	ND	ND	ND
18 : 1ω7*c* 11-methyl	ND	TR	TR	ND	ND	ND	ND	ND	ND
19 : 0 cyclo ω8*c*	TR	TR	TR	TR	ND	ND	TR	TR	TR
19 : 0 iso	ND	ND	ND	ND	ND	ND	ND	ND	TR
19 : 0 10-methyl	TR	ND	ND	ND	ND	ND	ND	ND	ND
Summed feature*									
2	ND	TR	TR	TR	TR	TR	ND	ND	ND
3	27.4	37.5	39.3	37.8	40.3	32.6	17.5	23.4	12.0
8	26.0	9.9	9.9	10.7	8.9	15.0	11.6	13.0	7.9

Values are percentages of total fatty acids. TR, Trace (<1 %); ND, not detected.

* summed feature 2, one or more of C12:0 aldehyde or unknown ECL (equivalent chain lengths) 10.928, isoC16:1 I and C14:0 3-OH; summed feature 3, $C_{16:1}\omega 7c$/$C_{16:1}\omega 6c$; summed feature 8, $C_{18:1}\omega 7c$/$C_{18:1}\omega 6c$.

Table S7. MIC range, MIC50 and MIC90 against the 42 isolates

Agent	MIC (μg/mL)			
	Breakpoint[a]	Range	MIC_{50}	MIC_{90}
Amikacin	64	1-8	2	4
Arbekacin	None	0.25-2	0.5	1
Aztreonam	32	8-256	64	128
Cefepime	None	2-16	4	16
Ceftazidime	32	1-16	4	16
Ciprofloxacin	None	<0.03125-0.5	0.0625	0.125
Colistin	4	0.25-4096	0.5	32
Gentamicin	16	0.25-2	0.5	2
Imipenem	8	0.25-8	1	4
Levofloxacin	8	<0.0625-2	0.5	1
Meropenem	8	0.125-16	2	8
Tigecycline	None	0.5-8	2	4
Tobramycin	16	0.25-2	0.5	1

[a]According to CLSI M100-S25 guideline.

Table S8. Detection of drug resistant genes in the 42 isolates

Isolate	Species	Resistant genes
BML-PP029	*P. otitidis*	$bla_{\text{POM-1}}$ like
BML-PP030^{T}	*P. parakoreensis* sp. nov.	*aadA6*
BML-PP033	*P. otitidis*	$bla_{\text{POM-1}}$ like
